# Impact of the COVID-19 pandemic on the complete rehabilitation journey of hip fracture patients in Italy: From surgical admission to rehabilitation facility discharge

**DOI:** 10.1371/journal.pone.0305966

**Published:** 2024-07-11

**Authors:** Heba Safwat Mhmoued Abdo Elhadidy, Gianfranco Politano, Roberta Onorati, Dario Catozzi, Maria Michela Gianino

**Affiliations:** 1 Department of Public Health Sciences and Pediatrics, Università di Torino, Torino, Italy; 2 Department of Control and Computer Engineering, Politecnico di Torino, Torino, Italy; 3 Regional Public Health Observatory, Epidemiology Unit, Local Healthcare Authority ASL TO3, Grugliasco, Italy; Policlinico Universitario A. Gemelli IRCCS - Universita Cattolica del Sacro Cuore Roma, ITALY

## Abstract

**Introduction:**

The COVID-19 pandemic led to a significant reorganization of health services, potentially affecting the quality of care for major public health concerns such as proximal femoral fractures. This study aimed to investigate potential changes in the timing of various steps in the patient journey after a hip fracture during the pandemic in Piedmont, a region in Northern Italy.

**Methods:**

A retrospective study was conducted on the discharge records of patients aged 65 or older who were admitted for hip surgery following a femur fracture in 2019 and 2020. The study examined four-time steps: duration from hospital admission to surgery, length of hospital stay, interval between hospital discharge and admission to the rehabilitation facility, and duration of stay at the rehabilitation facility. To mitigate biases linked to sex and age factors, groups well-balanced across 2019 and 2020 were created using propensity score estimation.

**Results:**

The dataset consisted of two cohorts of 583 patients each for the years 2019 and 2020. The average duration from admission to surgery was approximately 1.9 days in both years, with 75% of patients undergoing surgery within 2 days of hospital admission. The average hospital stay reduced from 13.49 days in 2019 to 11.34 days in 2020. The gap between hospital discharge and admission to rehabilitation was approximately 10–12 days, and the average duration of stay at the rehabilitation facility was about 31.6 days.

**Discussion:**

The study indicates that healthcare systems can exhibit resilience and adaptability, even during a global pandemic, to ensure high-quality and safe standards of care. However, further long-term studies are needed to fully understand the pandemic’s impact on primary health outcomes following hip replacement surgery and subsequent rehabilitation. The potential role of telemedicine in reducing the time between steps also warrants further investigation.

## Introduction

The COVID-19 pandemic has necessitated a swift reorganization of national health services to address emerging needs. In the majority of hospitals, elective surgeries were put on hold for several months, and operating theaters were repurposed as temporary intensive care units [1,2]. Concurrently, the activation of the emergency network for non-COVID-19 related reasons saw a significant decline. This has been attributed to a pervasive state of anxiety and fear, which deterred individuals from seeking medical assistance or led to delayed requests, even when necessary [3,4].

In this context, the widespread closure of non-essential services, coupled with lockdowns and other restrictions, led to a substantial decrease in the incidence of orthopedic trauma related to outdoor activities and transportation [5]. However, the reduction in major orthopedic trauma has only marginally affected accidental or low-energy fall trauma in the elderly, often associated with reduced bone mass. The most common outcome of these incidents is the proximal femoral fracture (PFF) [5,6]. For these patients, early surgery, when indicated, was ensured even during the most critical stages of the pandemic. This is because excessive surgical delay is linked to higher mortality [6–8], a risk further amplified by the potential for infection in the hospital setting [9].

PFF is a traumatic injury predominantly observed in late adulthood, often associated with conditions of osteoporosis or osteopenia. It is estimated that only 8% of such fractures occur in individuals under the age of 65 [10]. A systematic review with meta-analysis conducted in 2002 reported an incidence of femoral fractures in Italy of 242 per 100,000 inhabitants [11]. A more recent study analyzing the trend of femoral fracture incidence in Italians over 65 highlighted a significant increase between the first and last year of analysis [10]. The focus on femoral fractures in the elderly is primarily due to its high short-term and medium-term mortality rates, which are 9.2% within a month, 33% after one year, and 56% after five years [10,12]. Moreover, it is important to consider an additional 30% of patients with residual permanent disability and a 40% with a loss of independent ambulation [12].

Given the vulnerability of these patients, the selection of a surgical treatment and its timely execution are crucial. Evidence clearly shows that early surgery reduces both mortality and the incidence of bedsores [13]. Post-surgical rehabilitation aims to restore motor functions and autonomy prior to trauma. Early mobilization also mitigates complications related to prolonged bed rest [6]. While early mobilization is a marker of healthcare quality, the optimal timing and intensity to achieve this goal have not yet been established [14]. Longitudinal studies have shown that the functional status of patients with hip fractures deteriorates following a reduction in rehabilitation services [15,16].

We believe it is crucial to examine the impact of the COVID-19 pandemic on the quality of care provided by the Italian healthcare system for conditions of significant public concern, such as hip fractures. To the best of our knowledge, while some studies have evaluated the differences in the timeliness of hip fracture surgery, no study has analyzed the changes that occurred during the pandemic period in the entire journey after a hip fracture, from admission to the orthopedic ward to discharge from the rehabilitation facility. Therefore, the primary objective of this study was to investigate how the timing between these multiple steps changed before (2019) and during (2020) the pandemic period.

## Methods

A retrospective study was conducted on patients aged 65 years or older who were admitted to the hospital for hip surgery following a femur fracture in 2019 and 2020. The study was carried out in Piedmont, a region in northern Italy with nearly 3.3 million residents, 26% of whom are aged 65 years or older. This region was significantly impacted by the early spread of the pandemic in Italy. The reason for choosing the year 2020 for the study is that it represented the peak of the pandemic, which had a significant impact on healthcare services. The year 2019 served as a pre-pandemic baseline. The study period was selected as March, April, and May for both years to maximize the likelihood of detecting potential changes in rehabilitation pathways due to the altered context during the pandemic. Data were accessed on the 10^th^ of December 2022.

Hospital discharge records of all patients admitted with a primary or secondary diagnosis of an upper femur fracture were collected. The diagnosis was defined according to the International Classification of Diseases, 9th revision—Clinical Modification (ICD-9-CM code 820) [17], which is currently used in Italy. Exclusion criteria were established in line with the indicators specified by the “*Programma Nazionale Esiti*” [18], a tool for measuring, analyzing, evaluating, and monitoring the clinical and care performance of healthcare facilities. These criteria included: age under 65; non-resident in Italy; non-urgent hospital admission; daytime hospital care, known in Italy as “day hospital”; therapeutic or rehabilitative care [19]; polytrauma (diagnosis-related group, DRG 484–487); diagnosis or medical history of malignant tumors (primary/secondary ICD-9-CM codes 140.0–208.9, 238.6, V10); death within one day of hospitalization without hip fracture surgery. Additionally, patients who passed away after the rehabilitation path were excluded.

The hospital discharge records of all patients admitted with a primary or secondary diagnosis of an upper femur fracture were linked with the rehabilitation facility discharge records. The collected variables included: sex; age grouped into three categories (65–75, 76–85, ≥86 years); category of rehabilitation facility (public, accredited private, equivalent); and setting of the rehabilitation discharge (home, nursing home, re-hospitalization, or death during rehabilitation).

The Italian healthcare system is characterized by a mix of public and private providers. Public hospitals and clinics are the backbone of the system, offering a wide range of medical services, including orthopedic and rehabilitative care. These facilities are directly managed by the National Health Service. In addition to public healthcare infrastructure, Italy also has a growing private healthcare sector, which includes other types of rehabilitation service providers: accredited private facilities and equivalent facilities. Accreditation grants the status of “eligible facility to provide services on behalf of the National Health Service”. To achieve accreditation, private health facilities must meet safety and quality standards as per the different Regional Accreditation Laws. Furthermore, private university polyclinics, Scientific Institutes for Research, Hospitalization and Healthcare (IRCCS), and ecclesiastical institutions providing hospital care are considered health facilities equivalent to public ones, although they are not directly managed by the National Health Service.

In Italy, the care process for patients with femoral fractures typically involves admission to a hospital, surgical intervention, and a period of post-operative rehabilitation. This process is managed through a coordinated effort between the discharging hospital and the receiving rehabilitation facility, designed to ensure a seamless transition for the patient and minimize any potential gap between discharge and rehabilitation admission. However, factors such as bed availability, patient preference, or logistical issues may occasionally result in a delay.

### Statistical analysis

To delineate the rehabilitation path, we examined four-time steps, measured in days:

1. The duration from hospital admission to surgery;
2. The length of hospital stay, from admission to discharge;
3. The interval between hospital discharge and admission to the rehabilitation facility;
4. The duration of stay at the rehabilitation facility (rehabilitation time).

Each of these time steps represents a critical phase in the patient’s journey from injury to recovery. They were specifically chosen because they are managed by different teams, wards, or facilities, thereby providing a comprehensive view of the entire rehabilitation process.

A sub-level analysis of all collected variables was conducted for each time step. Mean values were compared parametrically using Welch’s t-test, with confidence intervals set at 95% (95% CI). In the first step, we also evaluated the proportion of cases with timely hip fracture surgery, defined as any of the following procedures initiated within two calendar days of hospital admission: closed reduction of fracture with internal fixation (ICD-9-CM codes 79.10, 79.15); open reduction of fracture with internal fixation (ICD-9-CM codes 79.30, 79.35); total or partial hip replacement (ICD-9-CM codes 81.51, 81.52).

All analyses were executed in a custom pipeline developed with R version 4.3.1. We utilized the “*MatchIT*” package for dataset balancing via propensity score estimation. This methodology facilitated the smoothing of biases related to sex and age factors across the years 2019 and 2020. This was achieved by creating well-balanced groups, thereby reducing selection biases, enhancing estimates, and mitigating confounding effects, resulting in a refined balance of covariates between the 2019 and 2020 cohorts. For this task, we employed the “*MatchIT*” package with the “*nearest*” method and “*glm*” adapted distance. Subsequently, we used the “*ggstatsplot*” package, an extension of the “*ggplot2*” package, to compute details of statistical tests. These were configured as parametric tests featuring pairwise comparisons and Bonferroni-adjusted p-values to yield a more robust estimation of treatment effects.

### Ethics

All data were obtained from the Health Information System of the Piedmont Region, derived from mandatory administrative health records. This system complies with regional, national, and European personal data protection legislation and supports the evaluation and monitoring activities of the Piedmont Region. There are no direct identifiers; data is accessed using a universal anonymous patient identification number. This number is a unique, irreversible code assigned prior to storage, enabling data management by accredited institutions without additional authorization. Data processing was exclusively conducted by the Regional Public Health Observatory (*Servizio Sovrazonale di Epidemiologia*, SEPI—Local Healthcare Authority ASL TO3), which handles anonymized data in accordance with the regional regulation (*Deliberazione della Giunta Regionale*, DGR January 10th 2012, n. 3–3259 –“Disciplinary of the modalities of access to the regional health information assets”).

We utilized statistical data in accordance with the deontological standards for processing National Statistical System data for research, as per art. 5 ter of Legislative Decree 33/2013, amended by Legislative Decree 97/2016 and 101/2018. Given the fully anonymized nature of the administrative ministerial data, Ethics Committee approval was not required.

## Results

### Sample characteristics

Upon balancing the dataset, we identified two cohorts of 583 patients each, with an equal distribution of gender and age for the years 2019 and 2020. The cohorts consisted of 471 women (80.79%) and 112 men (19.21%). Age-wise, 114 patients (19.55%) were in the 64–75 age group, 267 patients (45.80%) were in the 76–85 age group, and 202 patients (34.65%) were in the age group of 86 and above.

Post hip surgery, many patients were referred to an accredited private rehabilitation facility, accounting for 311 cases (53.35%) in 2019 and 318 cases (54.55%) in 2020. Other rehabilitation settings included equivalent facilities, accommodating 145 patients (24.87%) in both 2019 and 2020, and public facilities, with 127 patients (21.78%) in 2019 and 120 patients (20.58%) in 2020. Upon discharge from the rehabilitation facilities, most patients were sent home.

Specific per-year data are presented in *Table 1*.

**Table 1:** Cohort characteristics per year of study.

	2019, n (%)	2020, n (%)
**Rehabilitation facility** Public facility (ward of the same hospital / other facility) Equivalent facility Accredited private facility	127 (21.78%) 145 (24.87%) 311 (53.35%)	120 (20.58%) 145 (24.87%) 318 (54.55%)
**Rehabilitation discharge** Home Nursing home Re-hospitalization Death	525 (90.05%) 16 (2.74%) 39 (6.69%) 3 (0.52%)	445 (76.33%) 34 (5.83%) 84 (14.41%) 20 (3.43%)

### Timing analysis

The average duration between admission and surgery was 1.89 days (SD 1.81) in 2019 and 1.96 days (SD 2.63) in 2020, with a p-value of 0.60. In both years of analysis, 75% of patients underwent surgery within 2 days of hospital admission. The average hospital stay was 13.49 days (SD 6.85) in 2019 and reduced to 11.34 days (SD 7.28) in 2020, with a significant p-value of <0.001. The gap between hospital discharge and admission to rehabilitation was approximately 10.15 days (SD 32.60) in 2019 and 12.09 days (SD 33.00) in 2020, with a p-value of 0.31. The average duration of stay at the rehabilitation facility was 31.57 days (SD 13.80) in 2019 and 31.74 days (SD 20.10) in 2020, with a p-value of 0.87.

### Sub-level analysis

No significant differences were observed in the distribution of gender, age, rehabilitation facility, and discharge setting concerning time from hospital admission to surgery, time between hospital discharge and rehabilitation admission, or duration of rehabilitation. The only exceptions were the time between hospital admission and surgery, and the duration of rehabilitation, which significantly decreased for patients undergoing rehabilitation in equivalent and public facilities.

On the other hand, several variables were associated with a reduced average length of hospitalization between 2019 and 2020. Both genders and older ages were associated with a shorter hospital stay. Patients discharged from the rehabilitation facility to home or those who passed away during rehabilitation had a shorter hospitalization in 2020 compared to 2019: 13.47 days (95% CI 12.88–14.07) vs 11.29 (95% CI 10.60–11.98), with a p-value <0.001, and 18.67 (95% CI 8.63–28.71) vs 9.45 (95% CI 7.82–11.08) days, with a p-value of 0.046, respectively.

Complete results are displayed in *Table 2*.

**Table 2:** Average 4-time steps in days per year of study.

	Step 1: Average time in days between hospital admission and surgery			Step 2: Average time in days for the hospital stay			Step 3: Average time in days between hospital discharge and rehabilitation facility admission			Step 4: Average time in days for the rehabilitation		
** **	**2019,** **mean** **(95% CI)**	**2020,** **mean** **(95% CI)**	** p** **value**	**2019,** **mean** **(95% CI)**	**2020,** **mean** **(95% CI)**	** p** **value**	**2019,** **mean** **(95% CI)**	**2020,** **mean** **(95% CI)**	** p** **value**	**2019,** **mean** **(95% CI)**	**2020,** **mean** **(95% CI)**	** p** **value**
**Gender** Women Men	1.91 (1.74–2.08) 1.82 (1.57–2.08)	2.01 (1.75–2.26) 1.77 (1.45–2.08)	0.53 0.79	13.35 (12.74–13.96) 14.06 (12.66–15.47)	11.35 (10.71–11.98) 11.34 (9.77–12.91)	**<0.001** **0.01**	10.88 (7.71–14.06) 7.05 (9.2–17.2)	12.44 (9.34–15.54) 13.0 (8.5–17.5)	0.49 0.26	31.82 (30.57–33.07) 30.51 (27.97–33.05)	32.25 (30.45–34.06) 29.57 (25.72–32.42)	0.70 0.69
**Age** 65–75 76–85 ≥ 86	1.86 (1.53–2.19) 1.97 (1.73–2.20) 1.81 (1.59–2.04)	2.44 (1.92–2.96) 1.76 (1.59–1.94) 1.96 (1.46–2.45)	0.06 0.17 0.60	11.85 (10.83–12.87) 13.68 (12.81–14.55) 14.15 (13.20–15.11)	11.53 (10.26–12.79) 11.81 (10.85–12.77) 10.63 (9.73–11.54)	0.69 **0.005** **<0.001**	4.11 (0.45–7.78) 13.01 (8.46–17.56) 9.76 (5.50–14.02)	10.39 (4.83–15.96) 14.36 (9.67–19.04) 10.06 (6.53–13.58)	0.06 0.69 0.92	29.89 (26.73–33.04) 31.62 (30.06–33.18) 32.45 (30.68–34.22)	31.63 (27.56–35.70) 31.94 (29.77–34.10) 31.53 (28.53–34.54)	0.50 0.81 0.61
**Rehabilitation facility** Public facility (ward of the same hospital /other facility) Equivalent facility Accredited private facility	2.09 (1.73–2.45) 1.92 (1.68–2.16) 1.80 (1.59–2.01)	2.45 (2.02–2.88) 1.57 (1.36–1.78) 1.96 (1.61–2.30)	0.21 **0.03** 0.44	13.85 (12.81–14.90) 11.82 (11.03–12.61) 14.12 (13.24–14.99)	11.95 (10.51–13.39) 9.44 (8.84–10.04) 11.98 (11.09–12.88)	**0.04** **<0.001** **<0.001**	12.17 (5.39–18.96) 8.55 (3.53–13.58) 10.06 (6.62–13.50)	7.55 (3.17–11.93) 9.93 (4.22–15.64) 14.79 (10.94–18.64)	0.26 0.72 0.07	28.68 (26.47–30.88) 32.04 (29.23–34.86) 32.53 (31.15–33.90)	21.90 (18.91–24.89) 32.57 (29.62–35.51) 35.07 (32.75–37.39)	**<0.001** 0.80 0.06
**Rehabilitation discharge** Home Nursing home Re-hospitalization Death	1.90 (1.74–2.06) 2.13 (1.04–3.21) 1.72 (1.29–2.15) 1.33 (-2.46–5.13)	1.99 (1.73–2.25) 1.94 (1.17–2.71) 1.83 (1.37–2.29) 1.85 (1.20–2.50)	0.56 0.77 0.72 0.63	13.47 (12.88–14.07) 14.62 (10.31–18.94) 12.79 (11.15–14.44) 18.67 (8.63–28.71)	11.29 (10.60–11.98) 12.21 (9.92–14.49) 11.75 (10.08–13.42) 9.45 (7.82–11.08)	**<0.001** 0.31 0.37 **0.046**	8.92 (6.28–11.55) 41.94 (7.65–76.22) 14.44 (3.41–25.46) 0.00 (0.00–0.00)	12.08 (8.91–15.25) 16.32 (6.20–26.45) 13.30 (6.11–20.49) 0.15 (-0.16–0.46)	0.13 0.15 0.86 0.33	31.43 (30.35–32.51) 45.69 (36.11–55.26) 28.15 (21.05–35.26) 24.33 (4.41–44.26)	33.59 (31.77–35.41) 37.21 (31.26–43.15) 21.99 (17.29–26.69) 22.20 (15.09–29.31)	0.05 0.13 0.15 0.73

Abbreviations: CI, Confidence Intervals.

## Discussion

To the best of our knowledge, this study is the first to examine the comprehensive journey of patients with hip fractures, estimating the duration of various stages from hospital admission to discharge from the rehabilitation facility.

Considering the significance of early surgery [6,8], a notable outcome was that the average time from hospital admission to surgery (step 1) was within the recommended 2 days of hospitalization in both 2019 and 2020. Although not statistically significant, this finding aligns with existing literature: numerous studies have shown that the quality of hospital care remained consistent between pandemic and non-pandemic periods, indicating that the average time from hospital admission to surgery was unaffected [20–23]. The proportion of timely surgeries for hip fractures remained steady before and during the pandemic, at around 75% in both years under study. No significant differences were found concerning patient characteristics. Despite the need for improvement in Piedmont, this result is noteworthy as the percentage of patients treated within two days has been shown to exceed the Italian average (69.7%) and is comparable to the European Union average (75.1%) [24]. This highlights the system’s ability to adapt healthcare delivery and maintain a high standard of care globally.

The main finding of this study was the reduction in the average duration of hospitalization (step 2) in 2020 compared to 2019, particularly for patients aged over 75 years. Additionally, patients who were discharged to their homes from the rehabilitation facility or those who passed away during rehabilitation exhibited a shorter hospital stay in 2020 compared to 2019.

The average duration of hospitalization decreased during the COVID-19 pandemic, with the most significant reduction observed in older patients. Specifically, this reduction was significant in the 76–85 and ≥86 age groups, in both women and men. Several papers support these results, showing a reduction in the length of stay during the pandemic [25,26], and highlighting that this decreasing trend, which began long before due to fast-track [27,28], appears to have been exacerbated during the pandemic period. A plausible explanation is that early discharge protocols were maximized during the pandemic due to the risk of *SARS-CoV-2* infection, especially considering the frailty of these patients [25].

Furthermore, the reduction in the average duration of hospitalization was significant in patients discharged home after rehabilitation or those who passed away in the rehabilitation facility. This finding can be explained by the varying clinical conditions of the patients. It is reasonable to assume that patients who passed away during rehabilitation had a higher number of comorbidities, such as cardiopathies, nephropathies, diabetes, obstructive vascular diseases, pneumopathies, etc.,[29] and were discharged quickly from the hospital due to fear of the virus. Conversely, patients who were discharged home after rehabilitation likely had better clinical conditions at the time of hip replacement procedures and received earlier and more intensive in-hospital rehabilitation, resulting in a shorter hospital stay.

Another significant finding, closely related to the previous one, pertains to the average time between hospital discharge and admission to rehabilitation (step 3). Although not statistically significant, a slight increase was observed, underscoring the maintenance of effective communication between the hospital and territorial services. This noteworthy result can be attributed to the organizational aspects of care. The Piedmont Region has implemented a protocol for managing the care pathway of patients with hip fractures (*Percorsi diagnostici terapeutici assistenziali*, PDTA), aiming to ensure continuity of care from the hospital to other territorial settings. This protocol stipulates early mobilization post-surgery (within 48 hours), a physiatric evaluation leading to the development of an Individual Rehabilitation Project, and rapid patient discharge from the acute care area to the rehabilitation setting identified by the Physiatrist, provided the patient’s clinical stability is sufficient. Conversely, in cases of clinical instability, the protocol prescribes transfer to intermediate care beds (long-term care settings) for multidisciplinary specialist management, closely functionally and operationally connected with the acute care area, followed by access to rehabilitation facilities. Our results suggest that the care plan involving acute hospitals, communities, rehabilitation hospitals, and long-term care facilities likely remained effective even during the pandemic period. These findings underscore the importance of a liaison critical path in healthcare systems to ensure positive outcomes, as highlighted by limited literature [30,31].

Interestingly, the average duration of rehabilitation (step 4) remained stable during the COVID-19 pandemic. A significant reduction was observed in patients transferred from a ward of the same hospital or those who underwent rehabilitation in another public facility. This finding aligns with observations in other countries, as reported in the literature [31]. It is plausible that this decrease resulted from the conversion of several wards for the treatment of patients with COVID-19, reducing the availability of rehabilitation treatments and consequently shortening the time per patient in order to accommodate more patients. It is also possible that home discharge played a role in shortening the rehabilitation time. Indeed, home discharge is recommended when rehabilitation needs are minimal and adequate welfare support (family members or other caregivers) is available, with timely activation of home or outpatient rehabilitation treatment [32]. Given the need, this setting is certainly preferable for elderly patients, in order to minimize the length of hospitalization and avoid negative cognitive repercussions. It is also important to consider that home discharge could have been facilitated by the prominent role of Italian families in the care process [33,34]. Indeed, relatives often provide extensive support as caregivers, which could have led to an early discharge to home, knowing that the patient would be in a safe environment. Surprisingly, the results showed the maintenance of the average time for rehabilitation in equivalent and private facilities. It is likely that such types of facilities, not directly managed by the public system, were less affected by the pandemic, with fewer suspensions of admissions to free up space for patients with COVID-19 and fewer reductions in beds.

## Strengths and limitations

The primary strength of this study lies in its relevance to all public healthcare systems, which are continually striving to ensure healthcare quality and safety, especially during crises. To the best of our knowledge, this is the first study to analyze the entire patient journey, from surgical admission to discharge from rehabilitation facilities.

However, several limitations exist. Firstly, this study is a single-region retrospective analysis, which may limit its generalizability. Despite this, the research was conducted in a large region of Northern Italy, which shares similar healthcare systems, epidemiological conditions, and regional health profiles with other European countries. Therefore, while caution should be exercised in directly applying these results to other contexts, they could serve as a useful benchmark for future analyses on rehabilitation pathways in regions with comparable healthcare systems.

A second limitation, common to all studies based on administrative data, is the lack of information on patients’ clinical conditions, including physical and cognitive function, severity of illness, activities of daily living and environmental factors. This absence hinders the correlation of the duration of the entire path and its steps with the severity of the clinical presentation.

Another weakness is that the time between multiple steps, particularly between hospital admission and surgery, and between hospital discharge and rehabilitation facility admission, can only be quantified in calendar days. This measurement method impedes the precise assessment of early surgery and postoperative rehabilitation achievements, which are considered key performance indicators by many international organizations, such as OECD, Eurostat, and WHO.

Lastly, the study does not distinguish between different surgical treatments. However, by ensuring that these treatments are balanced in both groups being compared, the reported timings should not exhibit significant biases.

## Conclusions

This study suggests that a high quality of care was maintained throughout the pandemic, with no discernible gaps in the continuum of care. This continuity was facilitated by the well-structured organization of the Piedmont health system, which manages patients from hospitalization to rehabilitation, ensuring regular communication between various structures and personnel. The existence of an organized and formalized pathway for patients with hip fractures could have aided in maintaining high-quality care. This assertion is corroborated by the fact that surgery within two days was reported by 75% of the population, a statistic that remained consistent over the years. The timing of surgery is influenced by numerous factors, including the surgical capacity of hospitals and inter-hospital flow and access (e.g., timely discharges that create hospital capacity for new patients). These factors appear to have been effectively managed during the pandemic period.

Our findings indicate that healthcare systems can exhibit resilience and adaptability, even during a global pandemic, to ensure the provision of high-quality and safe standards of care. However, further long-term studies are required to fully understand the impact of the COVID-19 pandemic on primary health outcomes following hip replacement surgery and subsequent rehabilitation. Additionally, the role of telemedicine in reducing the time between steps, through remote follow-up visits and remote supervised home-based exercise therapy, warrants further investigation.

## Supporting information

S1 Table Dataset. Data underlying the findings described in the manuscript.
(DOCX)
