# Supplementary material for: Impact of the COVID-19 pandemic on the complete rehabilitation journey of hip fracture patients in Italy: From surgical admission to rehabilitation facility discharge
Source: PLoS One. 2024 Jul 11;19(7):e0305966. doi: 10.1371/journal.pone.0305966 (PMC11238963; doi:10.1371/journal.pone.0305966)
Supplement: S1 Table — Data underlying the findings described in the manuscript. (DOCX) [file pone.0305966.s001.docx]

***S1 Table. Dataset.***

| **Gender** | **Age** | **Year** | **Rehabilitation facility** | **Rehabilitation discharge** | **Step 1** | **Step 2** | **Step 3** | **Step 4** |
| --- | --- | --- | --- | --- | --- | --- | --- | --- |
| F | ≥ 86 | 2019 | Accredited private facility | Home | 0 | 22 | 100 | 20 |
| F | ≥ 86 | 2019 | Equivalent facility | Home | 4 | 14 | 0 | 41 |
| F | ≥ 86 | 2019 | Accredited private facility | Home | 4 | 13 | 0 | 48 |
| F | ≥ 86 | 2019 | Equivalent facility | Home | 1 | 16 | 0 | 24 |
| F | ≥ 86 | 2019 | Accredited private facility | Nursing home | 0 | 14 | 0 | 40 |
| F | ≥ 86 | 2019 | Public facility | Home | 1 | 20 | 0 | 24 |
| F | ≥ 86 | 2019 | Accredited private facility | Home | 4 | 10 | 0 | 43 |
| F | ≥ 86 | 2019 | Equivalent facility | Home | 3 | 10 | 0 | 36 |
| F | ≥ 86 | 2019 | Accredited private facility | Home | 0 | 14 | 0 | 38 |
| F | ≥ 86 | 2019 | Accredited private facility | Home | 2 | 7 | 0 | 38 |
| F | ≥ 86 | 2019 | Accredited private facility | Nursing home | 2 | 7 | 226 | 42 |
| F | 65-75 | 2019 | Public facility | Home | 1 | 11 | 0 | 25 |
| F | ≥ 86 | 2019 | Public facility | Home | 3 | 9 | 0 | 17 |
| F | 65-75 | 2019 | Equivalent facility | Home | 1 | 12 | 0 | 59 |
| F | 76-85 | 2019 | Accredited private facility | Re-hospitalization | 1 | 8 | 0 | 13 |
| F | 76-85 | 2019 | Accredited private facility | Re-hospitalization | 1 | 8 | 14 | 31 |
| F | 76-85 | 2019 | Accredited private facility | Re-hospitalization | 1 | 8 | 46 | 7 |
| F | 65-75 | 2019 | Equivalent facility | Home | 1 | 9 | 0 | 23 |
| F | ≥ 86 | 2019 | Equivalent facility | Home | 2 | 11 | 0 | 23 |
| F | ≥ 86 | 2019 | Equivalent facility | Home | 0 | 13 | 0 | 42 |
| F | 76-85 | 2019 | Accredited private facility | Home | 1 | 34 | 0 | 41 |
| F | 76-85 | 2019 | Accredited private facility | Home | 1 | 8 | 0 | 29 |
| F | ≥ 86 | 2019 | Accredited private facility | Home | 8 | 19 | 0 | 41 |
| F | ≥ 86 | 2019 | Equivalent facility | Home | 8 | 19 | 41 | 16 |
| F | 65-75 | 2019 | Accredited private facility | Home | 13 | 25 | 0 | 37 |
| F | 76-85 | 2019 | Accredited private facility | Home | 1 | 11 | 0 | 42 |
| F | 76-85 | 2019 | Accredited private facility | Home | 1 | 11 | 141 | 10 |
| F | 76-85 | 2019 | Equivalent facility | Home | 1 | 11 | 151 | 21 |
| F | 76-85 | 2019 | Accredited private facility | Re-hospitalization | 1 | 6 | 55 | 95 |
| F | 76-85 | 2019 | Accredited private facility | Home | 1 | 6 | 158 | 39 |
| F | 76-85 | 2019 | Accredited private facility | Home | 2 | 15 | 0 | 44 |
| F | ≥ 86 | 2019 | Accredited private facility | Home | 2 | 9 | 0 | 38 |
| F | 76-85 | 2019 | Equivalent facility | Home | 1 | 7 | 0 | 33 |
| F | 76-85 | 2019 | Equivalent facility | Home | 2 | 11 | 0 | 21 |
| F | 65-75 | 2019 | Public facility | Home | 1 | 18 | 0 | 26 |
| F | 76-85 | 2019 | Accredited private facility | Re-hospitalization | 4 | 9 | 0 | 2 |
| F | 76-85 | 2019 | Accredited private facility | Home | 4 | 9 | 20 | 30 |
| F | 76-85 | 2019 | Accredited private facility | Home | 3 | 28 | 0 | 32 |
| F | 76-85 | 2019 | Equivalent facility | Home | 1 | 9 | 79 | 29 |
| F | 76-85 | 2019 | Accredited private facility | Home | 1 | 10 | 0 | 31 |
| F | 76-85 | 2019 | Accredited private facility | Home | 3 | 12 | 0 | 14 |
| F | 76-85 | 2019 | Accredited private facility | Home | 3 | 13 | 0 | 34 |
| F | 65-75 | 2019 | Public facility | Home | 4 | 10 | 0 | 29 |
| F | 76-85 | 2019 | Public facility | Home | 0 | 13 | 0 | 21 |
| F | 76-85 | 2019 | Public facility | Home | 2 | 11 | 0 | 19 |
| F | 76-85 | 2019 | Public facility | Home | 3 | 9 | 0 | 39 |
| F | 76-85 | 2019 | Accredited private facility | Home | 1 | 11 | 0 | 30 |
| M | ≥ 86 | 2019 | Equivalent facility | Home | 1 | 9 | 0 | 29 |
| F | ≥ 86 | 2019 | Accredited private facility | Home | 3 | 18 | 0 | 23 |
| F | ≥ 86 | 2019 | Accredited private facility | Home | 4 | 10 | 0 | 29 |
| F | ≥ 86 | 2019 | Accredited private facility | Home | 0 | 13 | 0 | 38 |
| F | ≥ 86 | 2019 | Accredited private facility | Home | 2 | 22 | 0 | 29 |
| F | ≥ 86 | 2019 | Equivalent facility | Home | 2 | 16 | 0 | 27 |
| F | 76-85 | 2019 | Public facility | Home | 6 | 16 | 0 | 28 |
| F | ≥ 86 | 2019 | Equivalent facility | Home | 1 | 21 | 0 | 21 |
| F | 65-75 | 2019 | Accredited private facility | Home | 0 | 14 | 0 | 57 |
| F | 76-85 | 2019 | Equivalent facility | Home | 1 | 15 | 0 | 30 |
| F | 65-75 | 2019 | Accredited private facility | Home | 2 | 11 | 0 | 39 |
| M | ≥ 86 | 2019 | Equivalent facility | Home | 0 | 11 | 0 | 43 |
| M | ≥ 86 | 2019 | Accredited private facility | Home | 2 | 12 | 0 | 6 |
| F | ≥ 86 | 2019 | Accredited private facility | Death | 1 | 23 | 0 | 25 |
| F | 76-85 | 2019 | Accredited private facility | Home | 14 | 41 | 0 | 32 |
| M | ≥ 86 | 2019 | Accredited private facility | Home | 2 | 13 | 0 | 43 |
| F | ≥ 86 | 2019 | Accredited private facility | Home | 2 | 15 | 0 | 39 |
| F | ≥ 86 | 2019 | Accredited private facility | Home | 2 | 16 | 0 | 60 |
| M | 76-85 | 2019 | Equivalent facility | Home | 4 | 12 | 0 | 43 |
| F | ≥ 86 | 2019 | Accredited private facility | Nursing home | 1 | 40 | 0 | 28 |
| F | 76-85 | 2019 | Equivalent facility | Home | 1 | 7 | 0 | 18 |
| F | ≥ 86 | 2019 | Accredited private facility | Home | 6 | 13 | 0 | 29 |
| F | 76-85 | 2019 | Equivalent facility | Home | 1 | 7 | 0 | 18 |
| F | 65-75 | 2019 | Equivalent facility | Home | 2 | 11 | 0 | 32 |
| F | 65-75 | 2019 | Accredited private facility | Home | 2 | 11 | 46 | 29 |
| F | ≥ 86 | 2019 | Accredited private facility | Home | 1 | 14 | 0 | 37 |
| F | 76-85 | 2019 | Public facility | Home | 2 | 17 | 0 | 29 |
| F | ≥ 86 | 2019 | Accredited private facility | Home | 1 | 10 | 0 | 29 |
| M | 76-85 | 2019 | Accredited private facility | Home | 1 | 7 | 0 | 18 |
| F | ≥ 86 | 2019 | Accredited private facility | Home | 1 | 7 | 0 | 43 |
| M | 76-85 | 2019 | Public facility | Home | 3 | 22 | 56 | 28 |
| F | ≥ 86 | 2019 | Accredited private facility | Home | 1 | 33 | 0 | 25 |
| F | 76-85 | 2019 | Accredited private facility | Home | 2 | 12 | 45 | 29 |
| M | 76-85 | 2019 | Public facility | Home | 2 | 18 | 1 | 41 |
| F | 76-85 | 2019 | Public facility | Home | 1 | 12 | 0 | 45 |
| F | 76-85 | 2019 | Public facility | Home | 1 | 12 | 48 | 49 |
| F | 76-85 | 2019 | Equivalent facility | Home | 1 | 7 | 0 | 34 |
| F | 76-85 | 2019 | Equivalent facility | Home | 1 | 12 | 0 | 36 |
| M | ≥ 86 | 2019 | Public facility | Home | 0 | 29 | 0 | 40 |
| F | 65-75 | 2019 | Accredited private facility | Home | 0 | 18 | 0 | 24 |
| F | 76-85 | 2019 | Accredited private facility | Re-hospitalization | 0 | 17 | 0 | 1 |
| F | 76-85 | 2019 | Accredited private facility | Home | 0 | 17 | 2 | 23 |
| F | ≥ 86 | 2019 | Accredited private facility | Home | 7 | 13 | 0 | 42 |
| F | ≥ 86 | 2019 | Public facility | Home | 1 | 7 | 0 | 25 |
| F | 76-85 | 2019 | Equivalent facility | Home | 1 | 11 | 0 | 29 |
| F | 76-85 | 2019 | Accredited private facility | Home | 3 | 21 | 70 | 34 |
| F | 76-85 | 2019 | Accredited private facility | Home | 3 | 21 | 104 | 12 |
| F | 76-85 | 2019 | Public facility | Home | 3 | 21 | 209 | 7 |
| F | ≥ 86 | 2019 | Accredited private facility | Home | 3 | 20 | 0 | 29 |
| F | 65-75 | 2019 | Accredited private facility | Home | 2 | 6 | 0 | 23 |
| F | 65-75 | 2019 | Accredited private facility | Home | 0 | 5 | 0 | 37 |
| M | 76-85 | 2019 | Public facility | Home | 2 | 14 | 0 | 22 |
| F | 76-85 | 2019 | Public facility | Home | 3 | 17 | 0 | 29 |
| F | ≥ 86 | 2019 | Accredited private facility | Home | 2 | 35 | 0 | 30 |
| F | 76-85 | 2019 | Equivalent facility | Home | 3 | 14 | 0 | 38 |
| F | 76-85 | 2019 | Accredited private facility | Home | 2 | 9 | 0 | 40 |
| F | 65-75 | 2019 | Equivalent facility | Home | 1 | 9 | 0 | 30 |
| F | ≥ 86 | 2019 | Public facility | Home | 1 | 21 | 0 | 42 |
| F | 76-85 | 2019 | Equivalent facility | Home | 2 | 9 | 0 | 30 |
| F | 76-85 | 2019 | Equivalent facility | Home | 3 | 10 | 0 | 29 |
| F | ≥ 86 | 2019 | Public facility | Home | 2 | 14 | 28 | 14 |
| F | 76-85 | 2019 | Equivalent facility | Home | 1 | 27 | 0 | 47 |
| M | ≥ 86 | 2019 | Accredited private facility | Home | 0 | 10 | 0 | 43 |
| F | 76-85 | 2019 | Accredited private facility | Home | 1 | 8 | 0 | 31 |
| M | 76-85 | 2019 | Accredited private facility | Home | 0 | 5 | 0 | 33 |
| F | 76-85 | 2019 | Accredited private facility | Home | 1 | 6 | 0 | 37 |
| M | ≥ 86 | 2019 | Public facility | Home | 5 | 12 | 0 | 23 |
| M | 76-85 | 2019 | Accredited private facility | Home | 3 | 10 | 0 | 28 |
| F | 65-75 | 2019 | Public facility | Home | 3 | 7 | 0 | 39 |
| M | 76-85 | 2019 | Equivalent facility | Re-hospitalization | 1 | 12 | 0 | 11 |
| M | 76-85 | 2019 | Equivalent facility | Home | 1 | 12 | 12 | 51 |
| F | ≥ 86 | 2019 | Accredited private facility | Re-hospitalization | 1 | 16 | 0 | 54 |
| F | ≥ 86 | 2019 | Accredited private facility | Home | 1 | 16 | 54 | 32 |
| F | 65-75 | 2019 | Public facility | Home | 1 | 9 | 0 | 28 |
| M | ≥ 86 | 2019 | Accredited private facility | Re-hospitalization | 4 | 22 | 0 | 39 |
| M | ≥ 86 | 2019 | Accredited private facility | Home | 4 | 22 | 39 | 16 |
| F | 76-85 | 2019 | Public facility | Home | 0 | 7 | 0 | 19 |
| F | 76-85 | 2019 | Accredited private facility | Home | 2 | 11 | 0 | 32 |
| M | 76-85 | 2019 | Equivalent facility | Home | 1 | 8 | 0 | 39 |
| F | ≥ 86 | 2019 | Accredited private facility | Home | 0 | 17 | 0 | 41 |
| F | 65-75 | 2019 | Accredited private facility | Home | 2 | 9 | 0 | 38 |
| F | 76-85 | 2019 | Public facility | Home | 1 | 11 | 0 | 14 |
| F | 76-85 | 2019 | Public facility | Home | 2 | 16 | 0 | 40 |
| F | 76-85 | 2019 | Accredited private facility | Home | 2 | 16 | 40 | 24 |
| F | 76-85 | 2019 | Accredited private facility | Home | 1 | 28 | 0 | 42 |
| F | 76-85 | 2019 | Accredited private facility | Home | 12 | 17 | 0 | 31 |
| F | ≥ 86 | 2019 | Accredited private facility | Re-hospitalization | 2 | 10 | 0 | 47 |
| F | ≥ 86 | 2019 | Accredited private facility | Home | 2 | 10 | 47 | 38 |
| F | 76-85 | 2019 | Accredited private facility | Home | 2 | 14 | 0 | 40 |
| F | 76-85 | 2019 | Accredited private facility | Home | 2 | 14 | 40 | 10 |
| F | 76-85 | 2019 | Accredited private facility | Home | 2 | 9 | 0 | 23 |
| M | ≥ 86 | 2019 | Accredited private facility | Home | 1 | 15 | 73 | 33 |
| M | 76-85 | 2019 | Accredited private facility | Re-hospitalization | 3 | 17 | 0 | 42 |
| M | 76-85 | 2019 | Accredited private facility | Home | 3 | 17 | 42 | 29 |
| F | 76-85 | 2019 | Public facility | Home | 12 | 24 | 0 | 59 |
| F | 76-85 | 2019 | Public facility | Home | 12 | 24 | 163 | 43 |
| F | 76-85 | 2019 | Public facility | Home | 2 | 33 | 0 | 47 |
| F | ≥ 86 | 2019 | Equivalent facility | Home | 1 | 11 | 0 | 44 |
| F | 65-75 | 2019 | Accredited private facility | Home | 2 | 7 | 0 | 51 |
| F | 65-75 | 2019 | Equivalent facility | Home | 2 | 11 | 0 | 25 |
| F | 76-85 | 2019 | Accredited private facility | Home | 1 | 9 | 0 | 40 |
| F | 65-75 | 2019 | Equivalent facility | Home | 5 | 10 | 0 | 29 |
| F | 76-85 | 2019 | Accredited private facility | Home | 0 | 23 | 0 | 38 |
| F | 76-85 | 2019 | Public facility | Nursing home | 1 | 3 | 26 | 35 |
| F | ≥ 86 | 2019 | Accredited private facility | Home | 3 | 12 | 0 | 39 |
| F | 76-85 | 2019 | Equivalent facility | Home | 1 | 9 | 0 | 22 |
| M | ≥ 86 | 2019 | Accredited private facility | Home | 3 | 9 | 0 | 30 |
| F | 76-85 | 2019 | Public facility | Home | 2 | 22 | 0 | 28 |
| F | 65-75 | 2019 | Equivalent facility | Home | 3 | 10 | 0 | 27 |
| F | 76-85 | 2019 | Public facility | Home | 2 | 11 | 0 | 46 |
| F | 65-75 | 2019 | Equivalent facility | Home | 2 | 9 | 0 | 23 |
| F | 65-75 | 2019 | Public facility | Home | 3 | 13 | 0 | 28 |
| F | 76-85 | 2019 | Public facility | Home | 2 | 12 | 0 | 48 |
| F | 76-85 | 2019 | Public facility | Home | 2 | 12 | 225 | 39 |
| F | 76-85 | 2019 | Equivalent facility | Home | 5 | 13 | 0 | 22 |
| F | ≥ 86 | 2019 | Equivalent facility | Home | 2 | 8 | 0 | 41 |
| F | 76-85 | 2019 | Public facility | Home | 2 | 16 | 0 | 29 |
| F | 65-75 | 2019 | Equivalent facility | Home | 3 | 12 | 0 | 21 |
| F | 65-75 | 2019 | Accredited private facility | Home | 2 | 13 | 0 | 31 |
| F | ≥ 86 | 2019 | Equivalent facility | Home | 1 | 10 | 0 | 25 |
| M | 76-85 | 2019 | Equivalent facility | Home | 2 | 12 | 0 | 17 |
| F | ≥ 86 | 2019 | Accredited private facility | Home | 0 | 21 | 0 | 39 |
| F | ≥ 86 | 2019 | Accredited private facility | Home | 2 | 9 | 0 | 7 |
| F | ≥ 86 | 2019 | Equivalent facility | Home | 2 | 9 | 7 | 52 |
| F | ≥ 86 | 2019 | Public facility | Home | 2 | 13 | 0 | 24 |
| F | 65-75 | 2019 | Accredited private facility | Home | 1 | 16 | 0 | 40 |
| F | 76-85 | 2019 | Public facility | Home | 4 | 11 | 0 | 22 |
| F | ≥ 86 | 2019 | Public facility | Home | 0 | 8 | 0 | 25 |
| F | 65-75 | 2019 | Equivalent facility | Home | 2 | 8 | 0 | 35 |
| F | 76-85 | 2019 | Accredited private facility | Home | 2 | 12 | 0 | 39 |
| F | ≥ 86 | 2019 | Accredited private facility | Home | 1 | 8 | 0 | 30 |
| M | 76-85 | 2019 | Public facility | Home | 2 | 9 | 0 | 15 |
| F | ≥ 86 | 2019 | Accredited private facility | Home | 1 | 15 | 0 | 43 |
| M | ≥ 86 | 2019 | Accredited private facility | Home | 2 | 19 | 0 | 26 |
| F | 76-85 | 2019 | Public facility | Home | 0 | 10 | 9 | 28 |
| F | 76-85 | 2019 | Accredited private facility | Home | 0 | 10 | 191 | 23 |
| M | ≥ 86 | 2019 | Accredited private facility | Home | 2 | 12 | 63 | 30 |
| F | ≥ 86 | 2019 | Accredited private facility | Home | 1 | 10 | 0 | 42 |
| F | 65-75 | 2019 | Public facility | Home | 1 | 8 | 0 | 28 |
| F | ≥ 86 | 2019 | Public facility | Home | 1 | 9 | 35 | 38 |
| F | ≥ 86 | 2019 | Accredited private facility | Home | 2 | 16 | 0 | 26 |
| F | ≥ 86 | 2019 | Accredited private facility | Home | 1 | 11 | 0 | 30 |
| F | 76-85 | 2019 | Accredited private facility | Home | 0 | 10 | 0 | 29 |
| F | 76-85 | 2019 | Public facility | Home | 1 | 12 | 0 | 16 |
| F | ≥ 86 | 2019 | Accredited private facility | Home | 2 | 8 | 0 | 32 |
| F | 76-85 | 2019 | Accredited private facility | Home | 1 | 11 | 0 | 49 |
| F | ≥ 86 | 2019 | Accredited private facility | Re-hospitalization | 1 | 9 | 0 | 34 |
| F | ≥ 86 | 2019 | Accredited private facility | Home | 1 | 9 | 44 | 40 |
| F | ≥ 86 | 2019 | Accredited private facility | Home | 11 | 31 | 0 | 38 |
| F | ≥ 86 | 2019 | Accredited private facility | Home | 3 | 17 | 0 | 28 |
| F | 65-75 | 2019 | Accredited private facility | Home | 0 | 7 | 0 | 44 |
| M | ≥ 86 | 2019 | Public facility | Home | 4 | 16 | 0 | 21 |
| M | 76-85 | 2019 | Public facility | Re-hospitalization | 0 | 7 | 0 | 25 |
| F | ≥ 86 | 2019 | Equivalent facility | Home | 3 | 10 | 0 | 49 |
| F | ≥ 86 | 2019 | Accredited private facility | Home | 1 | 12 | 0 | 30 |
| F | 76-85 | 2019 | Accredited private facility | Nursing home | 0 | 14 | 0 | 68 |
| F | ≥ 86 | 2019 | Accredited private facility | Home | 1 | 7 | 0 | 41 |
| M | 76-85 | 2019 | Equivalent facility | Home | 2 | 17 | 0 | 29 |
| F | ≥ 86 | 2019 | Equivalent facility | Home | 4 | 10 | 0 | 31 |
| F | ≥ 86 | 2019 | Accredited private facility | Home | 1 | 8 | 0 | 44 |
| F | ≥ 86 | 2019 | Accredited private facility | Home | 0 | 35 | 0 | 20 |
| F | 76-85 | 2019 | Equivalent facility | Home | 2 | 10 | 0 | 30 |
| F | ≥ 86 | 2019 | Accredited private facility | Home | 1 | 15 | 0 | 20 |
| F | 65-75 | 2019 | Accredited private facility | Home | 2 | 13 | 0 | 40 |
| F | 76-85 | 2019 | Accredited private facility | Home | 0 | 12 | 0 | 30 |
| F | 65-75 | 2019 | Accredited private facility | Home | 1 | 7 | 0 | 17 |
| F | 76-85 | 2019 | Equivalent facility | Re-hospitalization | 1 | 8 | 0 | 22 |
| F | 76-85 | 2019 | Equivalent facility | Home | 1 | 8 | 22 | 7 |
| M | ≥ 86 | 2019 | Public facility | Home | 1 | 13 | 0 | 10 |
| F | 76-85 | 2019 | Accredited private facility | Home | 0 | 23 | 0 | 43 |
| F | 65-75 | 2019 | Accredited private facility | Home | 2 | 8 | 0 | 35 |
| F | 76-85 | 2019 | Accredited private facility | Home | 1 | 15 | 0 | 39 |
| F | 76-85 | 2019 | Accredited private facility | Nursing home | 1 | 15 | 71 | 47 |
| F | 76-85 | 2019 | Accredited private facility | Nursing home | 1 | 15 | 118 | 24 |
| F | 65-75 | 2019 | Public facility | Home | 1 | 10 | 10 | 32 |
| F | 76-85 | 2019 | Public facility | Home | 2 | 12 | 0 | 29 |
| F | 76-85 | 2019 | Equivalent facility | Home | 2 | 13 | 0 | 21 |
| M | ≥ 86 | 2019 | Accredited private facility | Home | 3 | 21 | 0 | 42 |
| F | 65-75 | 2019 | Accredited private facility | Home | 2 | 14 | 0 | 35 |
| F | ≥ 86 | 2019 | Accredited private facility | Home | 1 | 15 | 0 | 43 |
| F | 76-85 | 2019 | Public facility | Home | 0 | 16 | 0 | 18 |
| F | ≥ 86 | 2019 | Accredited private facility | Home | 1 | 16 | 0 | 39 |
| F | 76-85 | 2019 | Public facility | Re-hospitalization | 6 | 19 | 0 | 32 |
| F | 76-85 | 2019 | Public facility | Home | 6 | 19 | 67 | 41 |
| M | ≥ 86 | 2019 | Equivalent facility | Home | 1 | 19 | 0 | 9 |
| F | 76-85 | 2019 | Public facility | Home | 1 | 9 | 0 | 31 |
| F | 76-85 | 2019 | Accredited private facility | Home | 1 | 12 | 0 | 43 |
| F | 65-75 | 2019 | Accredited private facility | Home | 7 | 18 | 0 | 35 |
| M | ≥ 86 | 2019 | Accredited private facility | Home | 3 | 11 | 0 | 30 |
| M | ≥ 86 | 2019 | Accredited private facility | Home | 2 | 24 | 0 | 39 |
| M | ≥ 86 | 2019 | Accredited private facility | Home | 4 | 18 | 0 | 42 |
| M | 76-85 | 2019 | Accredited private facility | Home | 3 | 18 | 0 | 55 |
| F | 76-85 | 2019 | Accredited private facility | Home | 8 | 23 | 0 | 42 |
| F | ≥ 86 | 2019 | Accredited private facility | Home | 2 | 12 | 0 | 19 |
| M | ≥ 86 | 2019 | Public facility | Re-hospitalization | 2 | 11 | 48 | 9 |
| F | 76-85 | 2019 | Public facility | Home | 2 | 12 | 0 | 28 |
| F | ≥ 86 | 2019 | Accredited private facility | Home | 1 | 14 | 0 | 30 |
| F | 76-85 | 2019 | Equivalent facility | Home | 1 | 10 | 0 | 25 |
| M | ≥ 86 | 2019 | Public facility | Home | 1 | 19 | 102 | 70 |
| M | ≥ 86 | 2019 | Accredited private facility | Home | 0 | 6 | 0 | 36 |
| F | 76-85 | 2019 | Accredited private facility | Home | 2 | 37 | 0 | 29 |
| M | 65-75 | 2019 | Accredited private facility | Home | 3 | 25 | 0 | 35 |
| F | 65-75 | 2019 | Accredited private facility | Home | 0 | 11 | 0 | 36 |
| F | ≥ 86 | 2019 | Equivalent facility | Re-hospitalization | 1 | 15 | 0 | 13 |
| F | ≥ 86 | 2019 | Equivalent facility | Re-hospitalization | 1 | 15 | 27 | 88 |
| F | ≥ 86 | 2019 | Equivalent facility | Re-hospitalization | 1 | 15 | 117 | 31 |
| F | ≥ 86 | 2019 | Equivalent facility | Re-hospitalization | 1 | 15 | 159 | 36 |
| F | ≥ 86 | 2019 | Equivalent facility | Home | 1 | 15 | 195 | 16 |
| M | 65-75 | 2019 | Accredited private facility | Home | 0 | 4 | 0 | 18 |
| F | ≥ 86 | 2019 | Accredited private facility | Home | 2 | 9 | 0 | 14 |
| M | ≥ 86 | 2019 | Accredited private facility | Home | 3 | 6 | 0 | 32 |
| F | 65-75 | 2019 | Equivalent facility | Home | 3 | 12 | 0 | 34 |
| F | ≥ 86 | 2019 | Public facility | Home | 0 | 17 | 0 | 18 |
| F | 76-85 | 2019 | Accredited private facility | Home | 1 | 10 | 0 | 41 |
| F | 76-85 | 2019 | Public facility | Home | 2 | 12 | 0 | 40 |
| M | 65-75 | 2019 | Equivalent facility | Home | 1 | 15 | 0 | 30 |
| F | 65-75 | 2019 | Equivalent facility | Re-hospitalization | 5 | 13 | 0 | 4 |
| F | 65-75 | 2019 | Equivalent facility | Home | 5 | 13 | 4 | 16 |
| M | ≥ 86 | 2019 | Accredited private facility | Home | 1 | 12 | 0 | 33 |
| M | ≥ 86 | 2019 | Accredited private facility | Home | 1 | 12 | 54 | 46 |
| F | ≥ 86 | 2019 | Equivalent facility | Home | 1 | 17 | 0 | 25 |
| F | ≥ 86 | 2019 | Accredited private facility | Home | 2 | 7 | 0 | 38 |
| F | ≥ 86 | 2019 | Accredited private facility | Home | 4 | 15 | 0 | 29 |
| F | 76-85 | 2019 | Equivalent facility | Home | 0 | 9 | 0 | 35 |
| F | 76-85 | 2019 | Equivalent facility | Home | 0 | 8 | 0 | 41 |
| F | ≥ 86 | 2019 | Accredited private facility | Home | 1 | 9 | 0 | 32 |
| F | ≥ 86 | 2019 | Accredited private facility | Re-hospitalization | 1 | 23 | 0 | 36 |
| F | ≥ 86 | 2019 | Accredited private facility | Home | 1 | 23 | 36 | 11 |
| F | 76-85 | 2019 | Public facility | Home | 0 | 23 | 0 | 42 |
| F | 76-85 | 2019 | Equivalent facility | Home | 2 | 41 | 0 | 20 |
| F | ≥ 86 | 2019 | Equivalent facility | Home | 1 | 10 | 0 | 28 |
| F | ≥ 86 | 2019 | Equivalent facility | Home | 3 | 15 | 0 | 45 |
| F | 65-75 | 2019 | Equivalent facility | Home | 1 | 29 | 0 | 40 |
| F | ≥ 86 | 2019 | Public facility | Home | 1 | 14 | 0 | 42 |
| F | 76-85 | 2019 | Accredited private facility | Home | 2 | 9 | 0 | 51 |
| F | 76-85 | 2019 | Accredited private facility | Home | 2 | 23 | 0 | 41 |
| F | 76-85 | 2019 | Accredited private facility | Home | 1 | 7 | 0 | 21 |
| F | 76-85 | 2019 | Public facility | Home | 1 | 7 | 110 | 15 |
| F | 65-75 | 2019 | Public facility | Home | 0 | 4 | 0 | 29 |
| F | 65-75 | 2019 | Public facility | Nursing home | 2 | 13 | 0 | 31 |
| M | 76-85 | 2019 | Accredited private facility | Home | 1 | 11 | 0 | 44 |
| F | 76-85 | 2019 | Accredited private facility | Home | 3 | 9 | 0 | 27 |
| M | 76-85 | 2019 | Accredited private facility | Home | 2 | 10 | 0 | 35 |
| M | ≥ 86 | 2019 | Accredited private facility | Death | 3 | 15 | 0 | 32 |
| F | 76-85 | 2019 | Public facility | Home | 1 | 14 | 0 | 57 |
| F | 76-85 | 2019 | Public facility | Home | 1 | 16 | 0 | 19 |
| M | ≥ 86 | 2019 | Public facility | Home | 3 | 23 | 0 | 35 |
| F | 76-85 | 2019 | Accredited private facility | Home | 0 | 9 | 0 | 46 |
| F | 65-75 | 2019 | Equivalent facility | Home | 3 | 10 | 0 | 22 |
| M | 76-85 | 2019 | Accredited private facility | Home | 1 | 10 | 0 | 23 |
| F | ≥ 86 | 2019 | Accredited private facility | Home | 0 | 6 | 0 | 30 |
| F | ≥ 86 | 2019 | Accredited private facility | Home | 0 | 6 | 118 | 44 |
| F | 76-85 | 2019 | Public facility | Home | 0 | 11 | 0 | 36 |
| M | 65-75 | 2019 | Accredited private facility | Home | 1 | 18 | 0 | 38 |
| F | 76-85 | 2019 | Public facility | Home | 1 | 14 | 0 | 23 |
| F | ≥ 86 | 2019 | Accredited private facility | Home | 2 | 24 | 0 | 29 |
| M | 76-85 | 2019 | Public facility | Home | 1 | 21 | 0 | 24 |
| F | 76-85 | 2019 | Accredited private facility | Home | 3 | 10 | 0 | 44 |
| F | 76-85 | 2019 | Public facility | Nursing home | 4 | 16 | 0 | 24 |
| F | 65-75 | 2019 | Public facility | Home | 1 | 11 | 0 | 20 |
| F | ≥ 86 | 2019 | Accredited private facility | Death | 0 | 18 | 0 | 16 |
| M | 76-85 | 2019 | Public facility | Home | 6 | 20 | 0 | 23 |
| M | 76-85 | 2019 | Accredited private facility | Home | 1 | 11 | 0 | 43 |
| F | 65-75 | 2019 | Public facility | Home | 2 | 12 | 0 | 14 |
| F | ≥ 86 | 2019 | Accredited private facility | Home | 0 | 7 | 0 | 40 |
| F | ≥ 86 | 2019 | Accredited private facility | Home | 1 | 11 | 0 | 45 |
| F | 76-85 | 2019 | Equivalent facility | Home | 3 | 20 | 0 | 29 |
| F | ≥ 86 | 2019 | Equivalent facility | Home | 2 | 9 | 0 | 21 |
| F | 76-85 | 2019 | Accredited private facility | Home | 1 | 17 | 0 | 40 |
| M | ≥ 86 | 2019 | Accredited private facility | Home | 2 | 9 | 0 | 44 |
| F | 76-85 | 2019 | Accredited private facility | Home | 1 | 13 | 0 | 25 |
| F | 76-85 | 2019 | Equivalent facility | Home | 2 | 12 | 0 | 30 |
| M | ≥ 86 | 2019 | Accredited private facility | Home | 1 | 10 | 0 | 42 |
| F | 76-85 | 2019 | Accredited private facility | Home | 1 | 10 | 0 | 45 |
| M | 76-85 | 2019 | Equivalent facility | Re-hospitalization | 3 | 10 | 0 | 25 |
| M | 65-75 | 2019 | Accredited private facility | Home | 1 | 9 | 0 | 13 |
| F | 65-75 | 2019 | Accredited private facility | Home | 1 | 10 | 0 | 40 |
| M | 76-85 | 2019 | Public facility | Home | 0 | 7 | 0 | 20 |
| F | ≥ 86 | 2019 | Accredited private facility | Home | 7 | 16 | 0 | 42 |
| F | ≥ 86 | 2019 | Accredited private facility | Home | 5 | 12 | 0 | 41 |
| M | 65-75 | 2019 | Equivalent facility | Home | 1 | 12 | 0 | 31 |
| F | 76-85 | 2019 | Equivalent facility | Home | 2 | 13 | 0 | 52 |
| F | ≥ 86 | 2019 | Accredited private facility | Home | 2 | 15 | 0 | 27 |
| F | 76-85 | 2019 | Public facility | Home | 1 | 12 | 0 | 22 |
| F | ≥ 86 | 2019 | Accredited private facility | Home | 1 | 9 | 0 | 30 |
| M | 65-75 | 2019 | Equivalent facility | Home | 1 | 7 | 0 | 42 |
| F | 76-85 | 2019 | Equivalent facility | Home | 0 | 6 | 0 | 37 |
| F | ≥ 86 | 2019 | Accredited private facility | Home | 1 | 4 | 0 | 36 |
| F | 76-85 | 2019 | Accredited private facility | Home | 1 | 7 | 0 | 30 |
| F | 76-85 | 2019 | Accredited private facility | Home | 2 | 9 | 119 | 38 |
| F | 65-75 | 2019 | Public facility | Home | 3 | 19 | 0 | 26 |
| F | 76-85 | 2019 | Accredited private facility | Re-hospitalization | 3 | 28 | 0 | 40 |
| F | 76-85 | 2019 | Accredited private facility | Home | 3 | 28 | 40 | 5 |
| F | ≥ 86 | 2019 | Accredited private facility | Home | 1 | 14 | 0 | 40 |
| F | ≥ 86 | 2019 | Accredited private facility | Home | 4 | 17 | 0 | 31 |
| M | 65-75 | 2019 | Accredited private facility | Home | 2 | 20 | 0 | 22 |
| F | 76-85 | 2019 | Accredited private facility | Home | 3 | 12 | 0 | 24 |
| F | 76-85 | 2019 | Public facility | Re-hospitalization | 3 | 14 | 0 | 52 |
| F | 76-85 | 2019 | Public facility | Re-hospitalization | 3 | 14 | 53 | 55 |
| F | 76-85 | 2019 | Accredited private facility | Home | 3 | 14 | 108 | 19 |
| F | 76-85 | 2019 | Accredited private facility | Home | 2 | 9 | 0 | 30 |
| F | 76-85 | 2019 | Accredited private facility | Re-hospitalization | 1 | 8 | 0 | 23 |
| F | 76-85 | 2019 | Accredited private facility | Home | 1 | 8 | 24 | 7 |
| F | 76-85 | 2019 | Accredited private facility | Home | 0 | 15 | 0 | 38 |
| F | ≥ 86 | 2019 | Equivalent facility | Home | 1 | 7 | 0 | 35 |
| F | 76-85 | 2019 | Accredited private facility | Home | 1 | 11 | 0 | 23 |
| F | 76-85 | 2019 | Accredited private facility | Home | 0 | 11 | 0 | 47 |
| M | ≥ 86 | 2019 | Accredited private facility | Nursing home | 2 | 10 | 0 | 83 |
| F | 76-85 | 2019 | Public facility | Home | 4 | 11 | 0 | 28 |
| F | 76-85 | 2019 | Equivalent facility | Home | 2 | 11 | 0 | 29 |
| M | 76-85 | 2019 | Equivalent facility | Home | 1 | 11 | 0 | 42 |
| M | 76-85 | 2019 | Accredited private facility | Home | 0 | 14 | 0 | 29 |
| M | 76-85 | 2019 | Accredited private facility | Home | 1 | 10 | 0 | 18 |
| M | ≥ 86 | 2019 | Accredited private facility | Home | 2 | 32 | 0 | 42 |
| F | 76-85 | 2019 | Equivalent facility | Home | 2 | 9 | 0 | 47 |
| F | ≥ 86 | 2019 | Accredited private facility | Home | 0 | 20 | 0 | 29 |
| F | ≥ 86 | 2019 | Equivalent facility | Home | 2 | 7 | 0 | 36 |
| F | 76-85 | 2019 | Public facility | Home | 1 | 23 | 0 | 27 |
| F | 65-75 | 2019 | Equivalent facility | Home | 0 | 11 | 0 | 30 |
| M | 76-85 | 2019 | Equivalent facility | Home | 1 | 8 | 0 | 49 |
| F | ≥ 86 | 2019 | Accredited private facility | Re-hospitalization | 1 | 9 | 0 | 31 |
| F | ≥ 86 | 2019 | Accredited private facility | Home | 1 | 9 | 31 | 11 |
| F | ≥ 86 | 2019 | Accredited private facility | Home | 0 | 14 | 0 | 42 |
| F | 65-75 | 2019 | Equivalent facility | Home | 1 | 13 | 0 | 19 |
| F | 65-75 | 2019 | Accredited private facility | Home | 3 | 16 | 0 | 36 |
| F | ≥ 86 | 2019 | Accredited private facility | Home | 0 | 24 | 0 | 38 |
| F | 76-85 | 2019 | Equivalent facility | Home | 3 | 21 | 0 | 21 |
| F | 76-85 | 2019 | Accredited private facility | Home | 0 | 10 | 0 | 33 |
| F | ≥ 86 | 2019 | Accredited private facility | Home | 2 | 18 | 0 | 28 |
| F | ≥ 86 | 2019 | Accredited private facility | Home | 1 | 9 | 0 | 30 |
| F | 65-75 | 2019 | Equivalent facility | Home | 2 | 9 | 0 | 21 |
| F | 76-85 | 2019 | Accredited private facility | Home | 2 | 11 | 0 | 42 |
| F | 65-75 | 2019 | Accredited private facility | Home | 1 | 26 | 0 | 30 |
| M | 65-75 | 2019 | Equivalent facility | Home | 4 | 12 | 0 | 28 |
| F | 65-75 | 2019 | Equivalent facility | Home | 0 | 5 | 0 | 37 |
| M | 65-75 | 2019 | Accredited private facility | Home | 4 | 8 | 0 | 28 |
| F | 76-85 | 2019 | Public facility | Home | 1 | 9 | 0 | 38 |
| F | ≥ 86 | 2019 | Equivalent facility | Home | 1 | 17 | 0 | 42 |
| F | ≥ 86 | 2019 | Accredited private facility | Home | 0 | 11 | 0 | 35 |
| F | ≥ 86 | 2019 | Accredited private facility | Home | 1 | 6 | 0 | 42 |
| F | 76-85 | 2019 | Accredited private facility | Re-hospitalization | 1 | 11 | 0 | 4 |
| F | 76-85 | 2019 | Accredited private facility | Home | 1 | 11 | 16 | 40 |
| F | 76-85 | 2019 | Equivalent facility | Home | 3 | 11 | 0 | 42 |
| F | 76-85 | 2019 | Accredited private facility | Home | 1 | 18 | 0 | 24 |
| F | 76-85 | 2019 | Accredited private facility | Home | 2 | 6 | 0 | 28 |
| F | 76-85 | 2019 | Accredited private facility | Home | 1 | 19 | 0 | 30 |
| F | 76-85 | 2019 | Equivalent facility | Home | 2 | 9 | 0 | 46 |
| M | 76-85 | 2019 | Accredited private facility | Home | 2 | 19 | 0 | 34 |
| F | ≥ 86 | 2019 | Equivalent facility | Home | 0 | 7 | 0 | 48 |
| F | ≥ 86 | 2019 | Equivalent facility | Home | 0 | 7 | 48 | 11 |
| F | ≥ 86 | 2019 | Accredited private facility | Home | 3 | 27 | 0 | 46 |
| F | ≥ 86 | 2019 | Accredited private facility | Home | 3 | 27 | 46 | 8 |
| F | 76-85 | 2019 | Public facility | Home | 2 | 14 | 0 | 30 |
| F | 65-75 | 2019 | Accredited private facility | Home | 1 | 8 | 0 | 18 |
| M | 76-85 | 2019 | Accredited private facility | Home | 2 | 27 | 0 | 42 |
| M | 65-75 | 2019 | Accredited private facility | Home | 2 | 8 | 0 | 10 |
| F | 76-85 | 2019 | Public facility | Home | 1 | 11 | 0 | 34 |
| F | 76-85 | 2019 | Accredited private facility | Home | 2 | 9 | 0 | 27 |
| F | ≥ 86 | 2019 | Accredited private facility | Home | 2 | 26 | 0 | 31 |
| F | 76-85 | 2019 | Public facility | Home | 2 | 36 | 0 | 19 |
| F | 76-85 | 2019 | Public facility | Home | 2 | 36 | 189 | 28 |
| M | 65-75 | 2019 | Public facility | Re-hospitalization | 0 | 8 | 0 | 33 |
| M | 65-75 | 2019 | Accredited private facility | Home | 0 | 8 | 33 | 16 |
| F | 76-85 | 2019 | Public facility | Home | 1 | 10 | 0 | 33 |
| F | 65-75 | 2019 | Public facility | Home | 0 | 7 | 0 | 53 |
| F | ≥ 86 | 2019 | Accredited private facility | Home | 1 | 14 | 0 | 23 |
| F | 65-75 | 2019 | Accredited private facility | Home | 2 | 10 | 0 | 11 |
| F | ≥ 86 | 2019 | Accredited private facility | Home | 1 | 10 | 0 | 41 |
| F | 76-85 | 2019 | Equivalent facility | Home | 0 | 6 | 0 | 36 |
| F | 76-85 | 2019 | Accredited private facility | Home | 1 | 12 | 0 | 43 |
| F | 65-75 | 2019 | Equivalent facility | Home | 5 | 12 | 0 | 31 |
| F | ≥ 86 | 2019 | Accredited private facility | Home | 0 | 10 | 0 | 46 |
| M | 65-75 | 2019 | Accredited private facility | Home | 3 | 39 | 0 | 30 |
| F | 76-85 | 2019 | Equivalent facility | Home | 3 | 11 | 0 | 38 |
| F | 76-85 | 2019 | Accredited private facility | Home | 2 | 21 | 0 | 14 |
| F | 76-85 | 2019 | Accredited private facility | Home | 2 | 21 | 14 | 20 |
| F | ≥ 86 | 2019 | Accredited private facility | Home | 3 | 18 | 0 | 38 |
| F | ≥ 86 | 2019 | Equivalent facility | Home | 2 | 9 | 0 | 35 |
| M | 76-85 | 2019 | Accredited private facility | Home | 2 | 8 | 0 | 22 |
| M | 65-75 | 2019 | Accredited private facility | Home | 1 | 6 | 0 | 15 |
| M | 76-85 | 2019 | Accredited private facility | Home | 1 | 11 | 0 | 37 |
| F | ≥ 86 | 2019 | Public facility | Home | 2 | 13 | 0 | 18 |
| F | 76-85 | 2019 | Accredited private facility | Home | 2 | 11 | 0 | 61 |
| F | 76-85 | 2019 | Equivalent facility | Home | 3 | 7 | 0 | 33 |
| F | 76-85 | 2019 | Public facility | Home | 2 | 8 | 0 | 28 |
| F | 76-85 | 2019 | Accredited private facility | Home | 2 | 12 | 0 | 29 |
| F | ≥ 86 | 2019 | Accredited private facility | Home | 2 | 9 | 0 | 7 |
| F | 76-85 | 2019 | Public facility | Home | 1 | 7 | 0 | 48 |
| F | 76-85 | 2019 | Equivalent facility | Home | 2 | 8 | 0 | 31 |
| F | 65-75 | 2019 | Accredited private facility | Home | 2 | 9 | 0 | 20 |
| F | 65-75 | 2019 | Equivalent facility | Home | 1 | 7 | 0 | 29 |
| F | 65-75 | 2019 | Public facility | Home | 2 | 12 | 0 | 28 |
| F | ≥ 86 | 2019 | Accredited private facility | Home | 1 | 14 | 0 | 33 |
| M | 65-75 | 2019 | Public facility | Home | 1 | 13 | 0 | 11 |
| F | 76-85 | 2019 | Equivalent facility | Home | 1 | 5 | 0 | 8 |
| M | 76-85 | 2019 | Public facility | Home | 8 | 24 | 0 | 28 |
| F | 65-75 | 2019 | Accredited private facility | Home | 1 | 12 | 0 | 30 |
| M | 65-75 | 2019 | Accredited private facility | Re-hospitalization | 2 | 14 | 0 | 21 |
| M | 76-85 | 2019 | Public facility | Home | 2 | 19 | 0 | 14 |
| F | 65-75 | 2019 | Accredited private facility | Home | 9 | 14 | 0 | 16 |
| F | ≥ 86 | 2019 | Accredited private facility | Home | 3 | 19 | 0 | 48 |
| F | 65-75 | 2019 | Accredited private facility | Home | 2 | 11 | 0 | 27 |
| F | 76-85 | 2019 | Equivalent facility | Home | 7 | 16 | 0 | 26 |
| F | 65-75 | 2019 | Accredited private facility | Home | 2 | 14 | 0 | 31 |
| F | 76-85 | 2019 | Accredited private facility | Home | 0 | 7 | 0 | 26 |
| M | 65-75 | 2019 | Accredited private facility | Home | 2 | 14 | 0 | 32 |
| F | 76-85 | 2019 | Accredited private facility | Home | 1 | 64 | 0 | 42 |
| M | 76-85 | 2019 | Equivalent facility | Home | 3 | 14 | 0 | 46 |
| F | 76-85 | 2019 | Accredited private facility | Home | 1 | 12 | 0 | 32 |
| F | 76-85 | 2019 | Equivalent facility | Home | 3 | 13 | 0 | 37 |
| F | ≥ 86 | 2019 | Accredited private facility | Home | 1 | 15 | 0 | 18 |
| F | ≥ 86 | 2019 | Public facility | Home | 2 | 14 | 0 | 26 |
| F | 76-85 | 2019 | Accredited private facility | Home | 3 | 14 | 0 | 39 |
| F | 76-85 | 2019 | Public facility | Home | 1 | 18 | 0 | 17 |
| F | ≥ 86 | 2019 | Equivalent facility | Home | 3 | 9 | 0 | 29 |
| F | 65-75 | 2019 | Equivalent facility | Home | 1 | 15 | 0 | 30 |
| F | ≥ 86 | 2019 | Public facility | Home | 7 | 21 | 0 | 28 |
| F | 76-85 | 2019 | Accredited private facility | Home | 2 | 13 | 0 | 31 |
| F | ≥ 86 | 2019 | Accredited private facility | Home | 1 | 9 | 0 | 33 |
| M | 65-75 | 2019 | Accredited private facility | Home | 1 | 8 | 0 | 39 |
| F | 65-75 | 2019 | Accredited private facility | Re-hospitalization | 1 | 9 | 0 | 5 |
| F | 65-75 | 2019 | Accredited private facility | Home | 1 | 9 | 6 | 58 |
| F | 65-75 | 2019 | Accredited private facility | Home | 1 | 9 | 77 | 42 |
| F | 65-75 | 2019 | Accredited private facility | Home | 1 | 9 | 170 | 47 |
| F | 76-85 | 2019 | Accredited private facility | Home | 3 | 19 | 131 | 30 |
| F | 76-85 | 2019 | Accredited private facility | Home | 1 | 19 | 0 | 29 |
| F | ≥ 86 | 2019 | Equivalent facility | Home | 2 | 13 | 0 | 22 |
| F | ≥ 86 | 2019 | Accredited private facility | Home | 5 | 12 | 0 | 30 |
| F | 76-85 | 2019 | Public facility | Home | 1 | 8 | 0 | 14 |
| F | ≥ 86 | 2019 | Public facility | Home | 2 | 9 | 0 | 17 |
| F | 76-85 | 2019 | Accredited private facility | Home | 1 | 9 | 0 | 20 |
| M | ≥ 86 | 2019 | Accredited private facility | Home | 0 | 13 | 0 | 11 |
| F | ≥ 86 | 2019 | Public facility | Home | 2 | 8 | 0 | 28 |
| F | 65-75 | 2019 | Public facility | Home | 2 | 8 | 0 | 15 |
| F | 76-85 | 2019 | Equivalent facility | Home | 3 | 9 | 0 | 20 |
| M | 76-85 | 2019 | Equivalent facility | Home | 3 | 11 | 0 | 13 |
| F | 65-75 | 2019 | Accredited private facility | Home | 1 | 5 | 0 | 42 |
| F | 76-85 | 2019 | Accredited private facility | Home | 1 | 9 | 0 | 37 |
| F | ≥ 86 | 2019 | Accredited private facility | Home | 1 | 12 | 0 | 37 |
| F | 76-85 | 2019 | Accredited private facility | Home | 1 | 9 | 0 | 33 |
| M | 65-75 | 2019 | Equivalent facility | Home | 3 | 12 | 0 | 20 |
| F | 65-75 | 2019 | Public facility | Home | 1 | 12 | 71 | 22 |
| F | 76-85 | 2019 | Accredited private facility | Home | 1 | 9 | 0 | 28 |
| F | 76-85 | 2019 | Accredited private facility | Home | 3 | 8 | 0 | 31 |
| F | ≥ 86 | 2019 | Accredited private facility | Home | 2 | 8 | 0 | 30 |
| M | 65-75 | 2019 | Accredited private facility | Home | 0 | 10 | 0 | 23 |
| F | 76-85 | 2019 | Accredited private facility | Home | 1 | 8 | 0 | 36 |
| F | 76-85 | 2019 | Public facility | Home | 1 | 13 | 0 | 18 |
| F | 65-75 | 2019 | Equivalent facility | Home | 1 | 8 | 0 | 23 |
| F | 65-75 | 2019 | Equivalent facility | Home | 1 | 19 | 0 | 17 |
| F | ≥ 86 | 2019 | Public facility | Home | 1 | 9 | 0 | 35 |
| F | 76-85 | 2019 | Public facility | Home | 5 | 12 | 0 | 22 |
| F | 76-85 | 2019 | Equivalent facility | Home | 1 | 22 | 0 | 30 |
| F | 65-75 | 2019 | Accredited private facility | Home | 2 | 9 | 0 | 29 |
| F | ≥ 86 | 2019 | Public facility | Home | 2 | 17 | 0 | 14 |
| F | ≥ 86 | 2019 | Public facility | Home | 2 | 17 | 41 | 21 |
| M | 65-75 | 2019 | Accredited private facility | Home | 0 | 8 | 0 | 16 |
| F | 65-75 | 2019 | Public facility | Home | 2 | 11 | 0 | 23 |
| F | 76-85 | 2019 | Public facility | Home | 2 | 16 | 0 | 25 |
| F | 76-85 | 2019 | Accredited private facility | Home | 1 | 29 | 0 | 41 |
| F | ≥ 86 | 2019 | Accredited private facility | Re-hospitalization | 2 | 9 | 0 | 1 |
| F | ≥ 86 | 2019 | Accredited private facility | Home | 2 | 9 | 24 | 30 |
| F | 76-85 | 2019 | Accredited private facility | Home | 1 | 8 | 0 | 30 |
| M | ≥ 86 | 2019 | Equivalent facility | Home | 1 | 10 | 0 | 59 |
| F | 76-85 | 2019 | Equivalent facility | Home | 0 | 18 | 0 | 69 |
| M | ≥ 86 | 2019 | Accredited private facility | Home | 1 | 9 | 0 | 30 |
| F | 65-75 | 2019 | Equivalent facility | Home | 2 | 11 | 0 | 15 |
| F | 65-75 | 2019 | Equivalent facility | Home | 2 | 9 | 0 | 39 |
| F | 65-75 | 2019 | Accredited private facility | Home | 0 | 10 | 0 | 13 |
| M | 76-85 | 2019 | Equivalent facility | Home | 3 | 8 | 0 | 42 |
| M | 65-75 | 2019 | Equivalent facility | Home | 3 | 7 | 0 | 17 |
| F | 76-85 | 2019 | Accredited private facility | Home | 2 | 14 | 0 | 34 |
| F | ≥ 86 | 2019 | Public facility | Home | 2 | 15 | 0 | 15 |
| F | ≥ 86 | 2019 | Equivalent facility | Home | 0 | 7 | 0 | 35 |
| F | 76-85 | 2019 | Equivalent facility | Home | 4 | 10 | 0 | 30 |
| F | 65-75 | 2019 | Accredited private facility | Home | 1 | 11 | 0 | 42 |
| F | ≥ 86 | 2019 | Accredited private facility | Re-hospitalization | 2 | 11 | 0 | 40 |
| F | ≥ 86 | 2019 | Accredited private facility | Home | 1 | 16 | 0 | 18 |
| F | 76-85 | 2019 | Public facility | Home | 1 | 8 | 0 | 24 |
| F | 76-85 | 2019 | Public facility | Home | 1 | 8 | 55 | 17 |
| F | 65-75 | 2019 | Accredited private facility | Home | 0 | 8 | 0 | 22 |
| F | ≥ 86 | 2019 | Public facility | Home | 1 | 16 | 0 | 25 |
| F | 76-85 | 2019 | Public facility | Home | 1 | 10 | 0 | 21 |
| F | ≥ 86 | 2019 | Accredited private facility | Home | 3 | 13 | 0 | 49 |
| M | 76-85 | 2019 | Accredited private facility | Home | 3 | 14 | 0 | 38 |
| F | 76-85 | 2019 | Public facility | Home | 2 | 7 | 0 | 18 |
| F | 65-75 | 2019 | Accredited private facility | Home | 3 | 31 | 0 | 38 |
| M | 76-85 | 2019 | Accredited private facility | Home | 2 | 19 | 36 | 30 |
| M | 76-85 | 2019 | Accredited private facility | Home | 2 | 19 | 66 | 6 |
| F | 76-85 | 2019 | Accredited private facility | Home | 0 | 32 | 0 | 38 |
| M | ≥ 86 | 2019 | Accredited private facility | Home | 1 | 61 | 0 | 42 |
| F | 76-85 | 2019 | Public facility | Nursing home | 4 | 12 | 0 | 69 |
| F | 76-85 | 2019 | Public facility | Home | 8 | 20 | 0 | 75 |
| F | 76-85 | 2019 | Equivalent facility | Nursing home | 8 | 20 | 111 | 58 |
| M | 65-75 | 2019 | Equivalent facility | Re-hospitalization | 2 | 19 | 0 | 7 |
| M | 65-75 | 2019 | Equivalent facility | Nursing home | 2 | 19 | 52 | 34 |
| M | 76-85 | 2019 | Equivalent facility | Re-hospitalization | 1 | 8 | 0 | 3 |
| M | 76-85 | 2019 | Equivalent facility | Home | 1 | 8 | 10 | 43 |
| M | 65-75 | 2019 | Equivalent facility | Home | 2 | 11 | 0 | 31 |
| F | ≥ 86 | 2019 | Public facility | Home | 3 | 10 | 0 | 24 |
| F | ≥ 86 | 2019 | Accredited private facility | Home | 1 | 11 | 0 | 30 |
| F | 76-85 | 2019 | Equivalent facility | Home | 3 | 10 | 126 | 38 |
| M | 65-75 | 2019 | Equivalent facility | Home | 3 | 11 | 0 | 27 |
| F | 76-85 | 2019 | Accredited private facility | Home | 2 | 13 | 0 | 41 |
| F | ≥ 86 | 2019 | Public facility | Home | 0 | 7 | 0 | 24 |
| F | 76-85 | 2019 | Public facility | Home | 3 | 7 | 0 | 19 |
| F | 65-75 | 2019 | Public facility | Home | 3 | 20 | 0 | 28 |
| F | 76-85 | 2019 | Accredited private facility | Home | 2 | 12 | 0 | 49 |
| F | 76-85 | 2019 | Equivalent facility | Re-hospitalization | 1 | 8 | 0 | 28 |
| F | 76-85 | 2019 | Equivalent facility | Home | 1 | 8 | 35 | 23 |
| M | 76-85 | 2019 | Accredited private facility | Home | 0 | 14 | 0 | 42 |
| M | ≥ 86 | 2019 | Equivalent facility | Re-hospitalization | 1 | 18 | 0 | 44 |
| M | ≥ 86 | 2019 | Equivalent facility | Re-hospitalization | 1 | 18 | 44 | 14 |
| M | ≥ 86 | 2019 | Accredited private facility | Home | 1 | 18 | 58 | 39 |
| F | 65-75 | 2019 | Accredited private facility | Home | 2 | 13 | 0 | 41 |
| F | 76-85 | 2019 | Equivalent facility | Home | 3 | 12 | 0 | 21 |
| F | ≥ 86 | 2019 | Equivalent facility | Home | 2 | 14 | 0 | 36 |
| F | ≥ 86 | 2019 | Public facility | Home | 0 | 10 | 0 | 18 |
| F | ≥ 86 | 2019 | Accredited private facility | Nursing home | 4 | 17 | 67 | 59 |
| F | ≥ 86 | 2019 | Equivalent facility | Nursing home | 1 | 8 | 0 | 57 |
| F | ≥ 86 | 2019 | Accredited private facility | Home | 0 | 11 | 0 | 14 |
| F | 76-85 | 2019 | Equivalent facility | Home | 1 | 13 | 0 | 39 |
| F | 65-75 | 2019 | Equivalent facility | Home | 0 | 7 | 0 | 169 |
| F | 65-75 | 2019 | Public facility | Home | 2 | 9 | 0 | 21 |
| F | ≥ 86 | 2019 | Equivalent facility | Home | 3 | 11 | 0 | 35 |
| F | 76-85 | 2019 | Accredited private facility | Home | 2 | 30 | 0 | 41 |
| F | 76-85 | 2019 | Equivalent facility | Home | 5 | 12 | 0 | 31 |
| F | 65-75 | 2019 | Accredited private facility | Home | 0 | 12 | 0 | 29 |
| F | 76-85 | 2019 | Public facility | Home | 1 | 19 | 0 | 28 |
| F | ≥ 86 | 2019 | Accredited private facility | Home | 5 | 12 | 0 | 14 |
| F | 76-85 | 2019 | Public facility | Home | 2 | 17 | 0 | 20 |
| F | 76-85 | 2019 | Equivalent facility | Home | 2 | 12 | 0 | 33 |
| M | 76-85 | 2019 | Equivalent facility | Nursing home | 1 | 11 | 0 | 32 |
| F | ≥ 86 | 2019 | Accredited private facility | Home | 1 | 12 | 0 | 30 |
| F | 76-85 | 2019 | Accredited private facility | Home | 2 | 6 | 6 | 30 |
| M | 76-85 | 2019 | Accredited private facility | Home | 1 | 7 | 0 | 40 |
| F | 76-85 | 2020 | Accredited private facility | Death | 3 | 10 | 0 | 10 |
| F | ≥ 86 | 2020 | Equivalent facility | Home | 1 | 12 | 0 | 41 |
| F | 76-85 | 2020 | Equivalent facility | Home | 1 | 8 | 0 | 30 |
| F | 65-75 | 2020 | Public facility | Home | 2 | 9 | 0 | 17 |
| F | ≥ 86 | 2020 | Accredited private facility | Death | 1 | 5 | 0 | 42 |
| F | 76-85 | 2020 | Accredited private facility | Home | 4 | 29 | 27 | 72 |
| F | 76-85 | 2020 | Accredited private facility | Home | 1 | 4 | 0 | 50 |
| M | 76-85 | 2020 | Equivalent facility | Home | 7 | 13 | 0 | 23 |
| M | ≥ 86 | 2020 | Equivalent facility | Home | 2 | 8 | 0 | 35 |
| F | ≥ 86 | 2020 | Accredited private facility | Home | 2 | 7 | 127 | 21 |
| F | 76-85 | 2020 | Public facility | Home | 5 | 14 | 0 | 27 |
| M | 65-75 | 2020 | Equivalent facility | Home | 1 | 7 | 0 | 31 |
| M | 65-75 | 2020 | Equivalent facility | Home | 2 | 9 | 0 | 21 |
| F | 76-85 | 2020 | Accredited private facility | Home | 1 | 13 | 0 | 30 |
| F | ≥ 86 | 2020 | Accredited private facility | Home | 0 | 9 | 0 | 29 |
| F | ≥ 86 | 2020 | Equivalent facility | Home | 2 | 12 | 0 | 18 |
| F | 76-85 | 2020 | Accredited private facility | Home | 3 | 9 | 0 | 40 |
| F | 76-85 | 2020 | Accredited private facility | Home | 3 | 9 | 138 | 30 |
| F | 76-85 | 2020 | Equivalent facility | Home | 0 | 6 | 0 | 29 |
| F | 76-85 | 2020 | Equivalent facility | Home | 0 | 10 | 0 | 77 |
| F | ≥ 86 | 2020 | Equivalent facility | Home | 1 | 7 | 0 | 30 |
| F | ≥ 86 | 2020 | Public facility | Home | 2 | 9 | 0 | 21 |
| F | ≥ 86 | 2020 | Accredited private facility | Home | 1 | 13 | 0 | 40 |
| F | 76-85 | 2020 | Equivalent facility | Home | 3 | 6 | 0 | 17 |
| F | 76-85 | 2020 | Accredited private facility | Re-hospitalization | 3 | 13 | 0 | 67 |
| F | 76-85 | 2020 | Public facility | Re-hospitalization | 3 | 13 | 67 | 24 |
| F | 76-85 | 2020 | Accredited private facility | Home | 3 | 13 | 91 | 46 |
| F | 65-75 | 2020 | Equivalent facility | Home | 1 | 8 | 0 | 29 |
| F | 76-85 | 2020 | Equivalent facility | Death | 0 | 5 | 0 | 10 |
| F | ≥ 86 | 2020 | Equivalent facility | Re-hospitalization | 2 | 7 | 0 | 24 |
| F | 76-85 | 2020 | Accredited private facility | Home | 4 | 8 | 103 | 61 |
| F | 76-85 | 2020 | Public facility | Re-hospitalization | 7 | 21 | 0 | 21 |
| F | ≥ 86 | 2020 | Public facility | Nursing home | 2 | 9 | 0 | 27 |
| M | 76-85 | 2020 | Accredited private facility | Home | 2 | 6 | 202 | 14 |
| F | 76-85 | 2020 | Accredited private facility | Home | 1 | 10 | 0 | 38 |
| M | 65-75 | 2020 | Public facility | Home | 2 | 7 | 0 | 24 |
| M | 76-85 | 2020 | Accredited private facility | Home | 1 | 16 | 0 | 38 |
| F | 76-85 | 2020 | Accredited private facility | Home | 3 | 12 | 0 | 85 |
| F | 65-75 | 2020 | Accredited private facility | Death | 0 | 14 | 0 | 43 |
| F | ≥ 86 | 2020 | Accredited private facility | Nursing home | 1 | 11 | 0 | 39 |
| F | 65-75 | 2020 | Equivalent facility | Home | 4 | 11 | 0 | 47 |
| F | ≥ 86 | 2020 | Accredited private facility | Home | 2 | 35 | 0 | 34 |
| F | 76-85 | 2020 | Equivalent facility | Home | 1 | 9 | 0 | 36 |
| F | 76-85 | 2020 | Accredited private facility | Home | 1 | 8 | 0 | 71 |
| F | ≥ 86 | 2020 | Accredited private facility | Death | 2 | 9 | 0 | 20 |
| F | ≥ 86 | 2020 | Equivalent facility | Nursing home | 2 | 7 | 33 | 34 |
| F | 76-85 | 2020 | Equivalent facility | Home | 1 | 11 | 0 | 25 |
| F | 76-85 | 2020 | Accredited private facility | Home | 2 | 15 | 0 | 40 |
| F | 65-75 | 2020 | Equivalent facility | Home | 1 | 11 | 0 | 46 |
| F | ≥ 86 | 2020 | Public facility | Home | 9 | 18 | 0 | 27 |
| F | 76-85 | 2020 | Public facility | Home | 4 | 10 | 0 | 15 |
| F | 76-85 | 2020 | Equivalent facility | Home | 4 | 10 | 279 | 8 |
| F | ≥ 86 | 2020 | Public facility | Re-hospitalization | 2 | 9 | 0 | 9 |
| F | ≥ 86 | 2020 | Accredited private facility | Re-hospitalization | 3 | 9 | 0 | 1 |
| F | ≥ 86 | 2020 | Accredited private facility | Home | 3 | 9 | 2 | 37 |
| F | 65-75 | 2020 | Accredited private facility | Home | 0 | 8 | 0 | 41 |
| F | 65-75 | 2020 | Public facility | Re-hospitalization | 2 | 9 | 0 | 17 |
| F | ≥ 86 | 2020 | Equivalent facility | Home | 1 | 7 | 0 | 31 |
| F | 76-85 | 2020 | Accredited private facility | Home | 4 | 11 | 0 | 30 |
| F | ≥ 86 | 2020 | Accredited private facility | Home | 1 | 7 | 0 | 39 |
| F | ≥ 86 | 2020 | Accredited private facility | Home | 1 | 7 | 39 | 11 |
| F | 76-85 | 2020 | Accredited private facility | Home | 1 | 12 | 38 | 43 |
| M | ≥ 86 | 2020 | Public facility | Re-hospitalization | 4 | 9 | 0 | 23 |
| M | ≥ 86 | 2020 | Accredited private facility | Home | 4 | 9 | 23 | 20 |
| M | ≥ 86 | 2020 | Accredited private facility | Home | 4 | 9 | 43 | 17 |
| F | 76-85 | 2020 | Accredited private facility | Re-hospitalization | 0 | 21 | 0 | 14 |
| F | 76-85 | 2020 | Accredited private facility | Death | 3 | 13 | 0 | 23 |
| F | ≥ 86 | 2020 | Accredited private facility | Home | 0 | 3 | 18 | 43 |
| F | ≥ 86 | 2020 | Accredited private facility | Re-hospitalization | 1 | 15 | 0 | 37 |
| F | 65-75 | 2020 | Public facility | Re-hospitalization | 0 | 19 | 0 | 168 |
| F | 65-75 | 2020 | Accredited private facility | Re-hospitalization | 0 | 19 | 168 | 22 |
| F | 76-85 | 2020 | Public facility | Nursing home | 2 | 30 | 0 | 62 |
| F | 65-75 | 2020 | Public facility | Re-hospitalization | 1 | 8 | 0 | 5 |
| M | 76-85 | 2020 | Accredited private facility | Home | 2 | 10 | 0 | 36 |
| M | 76-85 | 2020 | Accredited private facility | Home | 2 | 10 | 36 | 11 |
| F | 65-75 | 2020 | Equivalent facility | Home | 0 | 9 | 0 | 28 |
| M | ≥ 86 | 2020 | Accredited private facility | Home | 1 | 7 | 0 | 31 |
| M | ≥ 86 | 2020 | Accredited private facility | Home | 1 | 15 | 86 | 17 |
| F | 76-85 | 2020 | Accredited private facility | Home | 1 | 17 | 1 | 29 |
| M | 76-85 | 2020 | Accredited private facility | Home | 1 | 27 | 0 | 40 |
| F | 76-85 | 2020 | Public facility | Home | 3 | 16 | 0 | 30 |
| F | ≥ 86 | 2020 | Accredited private facility | Re-hospitalization | 0 | 4 | 0 | 50 |
| F | ≥ 86 | 2020 | Public facility | Home | 0 | 4 | 72 | 16 |
| F | 76-85 | 2020 | Equivalent facility | Home | 1 | 11 | 0 | 23 |
| F | 76-85 | 2020 | Equivalent facility | Home | 1 | 5 | 0 | 61 |
| M | 65-75 | 2020 | Equivalent facility | Home | 2 | 13 | 0 | 58 |
| M | 76-85 | 2020 | Equivalent facility | Nursing home | 0 | 10 | 0 | 18 |
| M | 76-85 | 2020 | Accredited private facility | Home | 1 | 19 | 0 | 48 |
| F | ≥ 86 | 2020 | Equivalent facility | Re-hospitalization | 2 | 9 | 0 | 30 |
| F | ≥ 86 | 2020 | Equivalent facility | Re-hospitalization | 2 | 9 | 53 | 7 |
| F | 76-85 | 2020 | Public facility | Home | 2 | 15 | 0 | 21 |
| M | 76-85 | 2020 | Equivalent facility | Home | 1 | 12 | 0 | 25 |
| M | ≥ 86 | 2020 | Accredited private facility | Home | 2 | 10 | 0 | 41 |
| F | 65-75 | 2020 | Public facility | Home | 3 | 7 | 0 | 29 |
| F | ≥ 86 | 2020 | Accredited private facility | Home | 3 | 19 | 0 | 40 |
| F | 76-85 | 2020 | Accredited private facility | Home | 1 | 13 | 0 | 43 |
| F | 76-85 | 2020 | Public facility | Re-hospitalization | 1 | 7 | 0 | 10 |
| M | 76-85 | 2020 | Equivalent facility | Home | 1 | 7 | 0 | 73 |
| F | 65-75 | 2020 | Public facility | Home | 1 | 8 | 0 | 11 |
| F | ≥ 86 | 2020 | Accredited private facility | Home | 2 | 6 | 0 | 35 |
| F | 65-75 | 2020 | Equivalent facility | Home | 4 | 12 | 0 | 46 |
| F | 76-85 | 2020 | Accredited private facility | Home | 1 | 10 | 0 | 51 |
| F | ≥ 86 | 2020 | Equivalent facility | Home | 1 | 13 | 0 | 49 |
| F | 76-85 | 2020 | Accredited private facility | Home | 3 | 18 | 0 | 40 |
| F | 76-85 | 2020 | Accredited private facility | Home | 1 | 19 | 0 | 35 |
| F | 76-85 | 2020 | Accredited private facility | Home | 1 | 19 | 138 | 48 |
| F | 76-85 | 2020 | Accredited private facility | Home | 2 | 21 | 0 | 40 |
| M | 76-85 | 2020 | Accredited private facility | Home | 1 | 8 | 0 | 39 |
| F | 76-85 | 2020 | Accredited private facility | Home | 1 | 10 | 0 | 38 |
| M | 76-85 | 2020 | Accredited private facility | Home | 2 | 13 | 0 | 40 |
| F | 65-75 | 2020 | Accredited private facility | Home | 2 | 9 | 44 | 68 |
| M | ≥ 86 | 2020 | Accredited private facility | Home | 1 | 8 | 0 | 13 |
| F | 76-85 | 2020 | Accredited private facility | Home | 1 | 20 | 0 | 30 |
| F | ≥ 86 | 2020 | Public facility | Re-hospitalization | 2 | 12 | 0 | 9 |
| F | ≥ 86 | 2020 | Public facility | Home | 2 | 12 | 17 | 13 |
| M | ≥ 86 | 2020 | Accredited private facility | Home | 1 | 4 | 0 | 41 |
| F | 65-75 | 2020 | Accredited private facility | Death | 2 | 10 | 0 | 7 |
| F | 76-85 | 2020 | Public facility | Nursing home | 8 | 19 | 86 | 28 |
| F | 76-85 | 2020 | Equivalent facility | Home | 2 | 11 | 0 | 29 |
| M | 76-85 | 2020 | Accredited private facility | Re-hospitalization | 1 | 7 | 0 | 19 |
| M | 76-85 | 2020 | Public facility | Home | 1 | 7 | 49 | 17 |
| F | 76-85 | 2020 | Equivalent facility | Home | 1 | 14 | 0 | 32 |
| F | 65-75 | 2020 | Accredited private facility | Home | 1 | 44 | 0 | 36 |
| F | 65-75 | 2020 | Accredited private facility | Home | 3 | 7 | 0 | 15 |
| F | 76-85 | 2020 | Equivalent facility | Home | 0 | 18 | 0 | 18 |
| F | 76-85 | 2020 | Accredited private facility | Home | 4 | 12 | 0 | 44 |
| F | 76-85 | 2020 | Accredited private facility | Re-hospitalization | 2 | 14 | 0 | 21 |
| M | 76-85 | 2020 | Accredited private facility | Home | 1 | 12 | 0 | 32 |
| M | 76-85 | 2020 | Accredited private facility | Re-hospitalization | 3 | 10 | 5 | 3 |
| M | 76-85 | 2020 | Accredited private facility | Home | 3 | 10 | 8 | 20 |
| M | 76-85 | 2020 | Public facility | Home | 1 | 7 | 0 | 18 |
| F | ≥ 86 | 2020 | Accredited private facility | Home | 0 | 10 | 0 | 63 |
| F | ≥ 86 | 2020 | Accredited private facility | Home | 2 | 11 | 0 | 60 |
| M | ≥ 86 | 2020 | Equivalent facility | Home | 1 | 13 | 0 | 44 |
| F | 76-85 | 2020 | Accredited private facility | Home | 1 | 7 | 0 | 22 |
| F | 76-85 | 2020 | Equivalent facility | Home | 2 | 9 | 0 | 28 |
| F | 76-85 | 2020 | Public facility | Re-hospitalization | 1 | 9 | 0 | 4 |
| F | ≥ 86 | 2020 | Equivalent facility | Home | 1 | 8 | 0 | 31 |
| F | ≥ 86 | 2020 | Accredited private facility | Home | 3 | 7 | 0 | 40 |
| F | 76-85 | 2020 | Equivalent facility | Home | 3 | 10 | 0 | 24 |
| F | ≥ 86 | 2020 | Public facility | Home | 3 | 8 | 0 | 12 |
| F | 65-75 | 2020 | Accredited private facility | Home | 2 | 9 | 0 | 38 |
| F | 65-75 | 2020 | Accredited private facility | Home | 1 | 7 | 0 | 29 |
| F | ≥ 86 | 2020 | Accredited private facility | Home | 2 | 7 | 0 | 33 |
| F | 76-85 | 2020 | Accredited private facility | Home | 1 | 8 | 0 | 85 |
| F | 76-85 | 2020 | Accredited private facility | Home | 9 | 16 | 0 | 40 |
| M | ≥ 86 | 2020 | Accredited private facility | Home | 2 | 9 | 0 | 40 |
| F | ≥ 86 | 2020 | Equivalent facility | Home | 5 | 15 | 6 | 41 |
| F | 65-75 | 2020 | Equivalent facility | Home | 2 | 9 | 0 | 38 |
| M | ≥ 86 | 2020 | Accredited private facility | Home | 7 | 16 | 0 | 40 |
| F | 76-85 | 2020 | Accredited private facility | Home | 3 | 23 | 0 | 41 |
| F | 65-75 | 2020 | Equivalent facility | Home | 2 | 8 | 116 | 21 |
| F | 76-85 | 2020 | Accredited private facility | Home | 1 | 11 | 0 | 36 |
| F | 76-85 | 2020 | Accredited private facility | Death | 5 | 13 | 0 | 40 |
| F | ≥ 86 | 2020 | Accredited private facility | Home | 2 | 6 | 0 | 39 |
| F | 76-85 | 2020 | Equivalent facility | Home | 1 | 10 | 0 | 22 |
| F | 76-85 | 2020 | Equivalent facility | Re-hospitalization | 1 | 8 | 121 | 22 |
| F | 76-85 | 2020 | Equivalent facility | Home | 1 | 8 | 143 | 18 |
| F | 76-85 | 2020 | Accredited private facility | Re-hospitalization | 0 | 11 | 0 | 51 |
| F | ≥ 86 | 2020 | Equivalent facility | Home | 1 | 8 | 0 | 30 |
| F | 76-85 | 2020 | Public facility | Home | 2 | 8 | 0 | 16 |
| F | 65-75 | 2020 | Accredited private facility | Re-hospitalization | 3 | 11 | 11 | 25 |
| F | 65-75 | 2020 | Accredited private facility | Home | 3 | 11 | 36 | 40 |
| F | ≥ 86 | 2020 | Accredited private facility | Nursing home | 2 | 11 | 0 | 43 |
| F | 76-85 | 2020 | Accredited private facility | Home | 0 | 11 | 0 | 31 |
| F | 76-85 | 2020 | Accredited private facility | Home | 2 | 19 | 8 | 34 |
| F | ≥ 86 | 2020 | Accredited private facility | Home | 1 | 4 | 0 | 15 |
| F | 76-85 | 2020 | Accredited private facility | Home | 1 | 17 | 9 | 33 |
| F | ≥ 86 | 2020 | Public facility | Home | 2 | 10 | 0 | 22 |
| F | 65-75 | 2020 | Accredited private facility | Death | 1 | 9 | 0 | 31 |
| F | ≥ 86 | 2020 | Accredited private facility | Home | 2 | 6 | 0 | 49 |
| M | 65-75 | 2020 | Equivalent facility | Nursing home | 2 | 9 | 0 | 43 |
| F | 76-85 | 2020 | Accredited private facility | Home | 1 | 7 | 21 | 18 |
| F | 76-85 | 2020 | Accredited private facility | Home | 1 | 8 | 0 | 25 |
| F | 76-85 | 2020 | Accredited private facility | Home | 1 | 7 | 0 | 15 |
| F | 76-85 | 2020 | Accredited private facility | Home | 1 | 7 | 0 | 26 |
| F | 65-75 | 2020 | Accredited private facility | Home | 1 | 10 | 0 | 29 |
| F | ≥ 86 | 2020 | Accredited private facility | Home | 3 | 10 | 0 | 34 |
| F | 76-85 | 2020 | Accredited private facility | Home | 3 | 9 | 0 | 69 |
| M | 76-85 | 2020 | Equivalent facility | Re-hospitalization | 2 | 7 | 0 | 25 |
| F | 76-85 | 2020 | Accredited private facility | Nursing home | 3 | 11 | 0 | 65 |
| F | 76-85 | 2020 | Accredited private facility | Home | 3 | 11 | 93 | 42 |
| F | 76-85 | 2020 | Equivalent facility | Home | 2 | 8 | 0 | 30 |
| F | 76-85 | 2020 | Public facility | Home | 4 | 11 | 0 | 28 |
| M | 65-75 | 2020 | Accredited private facility | Death | 4 | 11 | 0 | 16 |
| F | 76-85 | 2020 | Public facility | Home | 3 | 15 | 0 | 27 |
| F | ≥ 86 | 2020 | Equivalent facility | Home | 2 | 9 | 0 | 30 |
| F | 76-85 | 2020 | Accredited private facility | Home | 1 | 7 | 0 | 36 |
| F | 76-85 | 2020 | Public facility | Home | 1 | 8 | 0 | 22 |
| F | 76-85 | 2020 | Public facility | Home | 6 | 14 | 0 | 22 |
| F | 76-85 | 2020 | Accredited private facility | Home | 1 | 8 | 0 | 30 |
| F | 76-85 | 2020 | Equivalent facility | Home | 1 | 13 | 0 | 28 |
| F | 65-75 | 2020 | Accredited private facility | Re-hospitalization | 2 | 9 | 0 | 8 |
| F | 65-75 | 2020 | Accredited private facility | Nursing home | 2 | 9 | 8 | 35 |
| F | 65-75 | 2020 | Public facility | Home | 2 | 8 | 0 | 19 |
| F | 76-85 | 2020 | Accredited private facility | Home | 0 | 7 | 0 | 58 |
| F | 65-75 | 2020 | Public facility | Re-hospitalization | 14 | 25 | 0 | 19 |
| F | 65-75 | 2020 | Accredited private facility | Home | 14 | 25 | 19 | 22 |
| M | ≥ 86 | 2020 | Accredited private facility | Home | 2 | 11 | 0 | 29 |
| F | 76-85 | 2020 | Equivalent facility | Home | 0 | 8 | 0 | 85 |
| M | 76-85 | 2020 | Accredited private facility | Home | 0 | 11 | 0 | 30 |
| F | ≥ 86 | 2020 | Accredited private facility | Home | 3 | 5 | 8 | 40 |
| F | 76-85 | 2020 | Accredited private facility | Home | 1 | 7 | 0 | 42 |
| F | 76-85 | 2020 | Accredited private facility | Home | 1 | 9 | 0 | 41 |
| F | 76-85 | 2020 | Accredited private facility | Home | 0 | 8 | 0 | 42 |
| F | 76-85 | 2020 | Public facility | Home | 4 | 11 | 0 | 30 |
| M | ≥ 86 | 2020 | Equivalent facility | Re-hospitalization | 3 | 6 | 0 | 31 |
| M | ≥ 86 | 2020 | Equivalent facility | Home | 3 | 6 | 31 | 23 |
| F | ≥ 86 | 2020 | Equivalent facility | Home | 1 | 9 | 0 | 24 |
| F | 76-85 | 2020 | Equivalent facility | Home | 2 | 7 | 0 | 47 |
| M | 76-85 | 2020 | Equivalent facility | Re-hospitalization | 1 | 8 | 0 | 13 |
| M | 76-85 | 2020 | Equivalent facility | Home | 1 | 8 | 16 | 42 |
| M | 76-85 | 2020 | Accredited private facility | Home | 1 | 8 | 110 | 3 |
| F | 76-85 | 2020 | Accredited private facility | Home | 2 | 10 | 0 | 39 |
| F | ≥ 86 | 2020 | Accredited private facility | Home | 1 | 5 | 0 | 36 |
| F | ≥ 86 | 2020 | Accredited private facility | Home | 4 | 11 | 0 | 43 |
| F | 76-85 | 2020 | Public facility | Home | 1 | 14 | 0 | 24 |
| F | 76-85 | 2020 | Accredited private facility | Re-hospitalization | 0 | 39 | 0 | 18 |
| F | 76-85 | 2020 | Public facility | Home | 0 | 39 | 47 | 14 |
| F | 76-85 | 2020 | Accredited private facility | Re-hospitalization | 2 | 7 | 0 | 8 |
| F | 76-85 | 2020 | Accredited private facility | Home | 2 | 7 | 14 | 40 |
| F | 65-75 | 2020 | Equivalent facility | Home | 1 | 6 | 0 | 23 |
| F | ≥ 86 | 2020 | Public facility | Death | 4 | 12 | 0 | 20 |
| F | ≥ 86 | 2020 | Equivalent facility | Home | 1 | 10 | 0 | 34 |
| F | 76-85 | 2020 | Public facility | Home | 3 | 8 | 0 | 21 |
| F | ≥ 86 | 2020 | Equivalent facility | Home | 2 | 6 | 0 | 19 |
| F | ≥ 86 | 2020 | Accredited private facility | Home | 2 | 32 | 0 | 22 |
| F | ≥ 86 | 2020 | Accredited private facility | Re-hospitalization | 1 | 10 | 13 | 32 |
| F | 76-85 | 2020 | Accredited private facility | Home | 1 | 14 | 0 | 29 |
| F | 65-75 | 2020 | Accredited private facility | Home | 1 | 8 | 0 | 32 |
| F | ≥ 86 | 2020 | Equivalent facility | Home | 0 | 14 | 18 | 45 |
| F | 76-85 | 2020 | Equivalent facility | Home | 0 | 6 | 0 | 46 |
| F | ≥ 86 | 2020 | Accredited private facility | Re-hospitalization | 0 | 11 | 7 | 28 |
| F | ≥ 86 | 2020 | Accredited private facility | Home | 0 | 11 | 35 | 211 |
| F | 76-85 | 2020 | Equivalent facility | Home | 0 | 9 | 0 | 33 |
| F | 76-85 | 2020 | Accredited private facility | Re-hospitalization | 0 | 9 | 166 | 16 |
| M | 65-75 | 2020 | Public facility | Home | 0 | 5 | 55 | 9 |
| F | ≥ 86 | 2020 | Accredited private facility | Home | 4 | 18 | 0 | 24 |
| F | ≥ 86 | 2020 | Equivalent facility | Home | 1 | 12 | 0 | 37 |
| F | 76-85 | 2020 | Accredited private facility | Home | 1 | 12 | 141 | 30 |
| F | 76-85 | 2020 | Accredited private facility | Home | 2 | 7 | 0 | 40 |
| M | 76-85 | 2020 | Equivalent facility | Home | 3 | 11 | 0 | 77 |
| F | 76-85 | 2020 | Equivalent facility | Home | 0 | 10 | 0 | 42 |
| F | ≥ 86 | 2020 | Equivalent facility | Home | 1 | 6 | 0 | 30 |
| F | ≥ 86 | 2020 | Accredited private facility | Home | 1 | 10 | 0 | 40 |
| F | 76-85 | 2020 | Accredited private facility | Home | 2 | 12 | 0 | 19 |
| F | 76-85 | 2020 | Accredited private facility | Home | 3 | 12 | 0 | 17 |
| F | 76-85 | 2020 | Equivalent facility | Home | 1 | 8 | 0 | 19 |
| F | 65-75 | 2020 | Equivalent facility | Home | 0 | 6 | 0 | 15 |
| F | 76-85 | 2020 | Accredited private facility | Nursing home | 1 | 19 | 0 | 29 |
| F | 76-85 | 2020 | Public facility | Home | 1 | 10 | 0 | 18 |
| F | 65-75 | 2020 | Public facility | Re-hospitalization | 6 | 12 | 0 | 19 |
| F | 65-75 | 2020 | Public facility | Home | 6 | 12 | 22 | 22 |
| F | ≥ 86 | 2020 | Accredited private facility | Home | 0 | 5 | 0 | 46 |
| F | ≥ 86 | 2020 | Accredited private facility | Re-hospitalization | 2 | 15 | 0 | 31 |
| F | ≥ 86 | 2020 | Accredited private facility | Home | 0 | 5 | 0 | 45 |
| F | 76-85 | 2020 | Public facility | Home | 1 | 7 | 0 | 29 |
| F | 76-85 | 2020 | Public facility | Home | 1 | 7 | 65 | 21 |
| F | 65-75 | 2020 | Public facility | Home | 2 | 32 | 0 | 19 |
| F | 65-75 | 2020 | Accredited private facility | Home | 1 | 14 | 0 | 30 |
| M | 76-85 | 2020 | Equivalent facility | Home | 0 | 12 | 0 | 30 |
| M | 76-85 | 2020 | Equivalent facility | Home | 0 | 5 | 0 | 45 |
| F | ≥ 86 | 2020 | Accredited private facility | Home | 0 | 8 | 0 | 69 |
| F | 76-85 | 2020 | Accredited private facility | Home | 3 | 17 | 0 | 73 |
| F | 76-85 | 2020 | Public facility | Home | 1 | 7 | 0 | 15 |
| F | ≥ 86 | 2020 | Accredited private facility | Home | 1 | 8 | 104 | 36 |
| F | ≥ 86 | 2020 | Accredited private facility | Home | 3 | 11 | 0 | 42 |
| M | 76-85 | 2020 | Accredited private facility | Home | 4 | 68 | 0 | 42 |
| M | 76-85 | 2020 | Equivalent facility | Home | 1 | 7 | 0 | 22 |
| F | ≥ 86 | 2020 | Accredited private facility | Home | 1 | 9 | 0 | 30 |
| F | ≥ 86 | 2020 | Accredited private facility | Home | 2 | 6 | 39 | 40 |
| F | 76-85 | 2020 | Equivalent facility | Home | 2 | 9 | 0 | 11 |
| M | ≥ 86 | 2020 | Equivalent facility | Home | 1 | 6 | 0 | 28 |
| F | ≥ 86 | 2020 | Equivalent facility | Home | 1 | 5 | 0 | 62 |
| F | ≥ 86 | 2020 | Accredited private facility | Home | 1 | 9 | 18 | 47 |
| F | 76-85 | 2020 | Public facility | Re-hospitalization | 2 | 7 | 0 | 5 |
| F | 76-85 | 2020 | Public facility | Home | 2 | 7 | 13 | 36 |
| F | 76-85 | 2020 | Accredited private facility | Home | 2 | 8 | 0 | 40 |
| F | 65-75 | 2020 | Public facility | Re-hospitalization | 8 | 10 | 0 | 59 |
| F | 65-75 | 2020 | Accredited private facility | Home | 8 | 10 | 59 | 40 |
| F | 76-85 | 2020 | Public facility | Home | 1 | 16 | 10 | 17 |
| F | 76-85 | 2020 | Accredited private facility | Home | 3 | 18 | 0 | 30 |
| F | ≥ 86 | 2020 | Accredited private facility | Home | 1 | 10 | 0 | 39 |
| F | 76-85 | 2020 | Public facility | Home | 2 | 6 | 0 | 25 |
| M | ≥ 86 | 2020 | Accredited private facility | Death | 1 | 7 | 0 | 33 |
| F | 65-75 | 2020 | Accredited private facility | Re-hospitalization | 2 | 16 | 0 | 6 |
| F | 65-75 | 2020 | Accredited private facility | Home | 2 | 16 | 64 | 40 |
| F | ≥ 86 | 2020 | Equivalent facility | Home | 0 | 13 | 0 | 28 |
| F | 65-75 | 2020 | Accredited private facility | Nursing home | 1 | 24 | 0 | 40 |
| F | 76-85 | 2020 | Equivalent facility | Home | 0 | 6 | 0 | 30 |
| F | ≥ 86 | 2020 | Equivalent facility | Home | 2 | 9 | 0 | 23 |
| F | 76-85 | 2020 | Public facility | Home | 3 | 8 | 0 | 17 |
| F | 65-75 | 2020 | Accredited private facility | Home | 15 | 33 | 0 | 95 |
| M | 76-85 | 2020 | Accredited private facility | Home | 1 | 23 | 0 | 3 |
| F | 65-75 | 2020 | Accredited private facility | Home | 4 | 19 | 0 | 42 |
| F | ≥ 86 | 2020 | Accredited private facility | Re-hospitalization | 1 | 10 | 0 | 13 |
| F | ≥ 86 | 2020 | Accredited private facility | Home | 1 | 10 | 13 | 27 |
| M | ≥ 86 | 2020 | Public facility | Home | 0 | 21 | 0 | 10 |
| F | 76-85 | 2020 | Accredited private facility | Re-hospitalization | 1 | 13 | 60 | 53 |
| F | 76-85 | 2020 | Accredited private facility | Home | 1 | 13 | 113 | 28 |
| F | 76-85 | 2020 | Accredited private facility | Home | 1 | 9 | 0 | 40 |
| F | 65-75 | 2020 | Equivalent facility | Home | 3 | 21 | 0 | 26 |
| F | 76-85 | 2020 | Equivalent facility | Home | 3 | 8 | 0 | 50 |
| F | 65-75 | 2020 | Public facility | Home | 2 | 9 | 0 | 17 |
| M | ≥ 86 | 2020 | Accredited private facility | Home | 4 | 12 | 0 | 23 |
| F | ≥ 86 | 2020 | Equivalent facility | Nursing home | 0 | 6 | 0 | 29 |
| F | 76-85 | 2020 | Accredited private facility | Home | 1 | 12 | 0 | 22 |
| F | 76-85 | 2020 | Equivalent facility | Home | 1 | 12 | 146 | 63 |
| F | 76-85 | 2020 | Public facility | Home | 3 | 10 | 0 | 22 |
| F | 76-85 | 2020 | Accredited private facility | Home | 3 | 5 | 27 | 43 |
| F | ≥ 86 | 2020 | Accredited private facility | Home | 2 | 9 | 0 | 20 |
| M | 76-85 | 2020 | Accredited private facility | Home | 0 | 5 | 0 | 16 |
| F | 76-85 | 2020 | Accredited private facility | Death | 2 | 7 | 0 | 7 |
| F | ≥ 86 | 2020 | Equivalent facility | Re-hospitalization | 1 | 9 | 0 | 27 |
| F | ≥ 86 | 2020 | Equivalent facility | Re-hospitalization | 1 | 9 | 38 | 5 |
| F | ≥ 86 | 2020 | Accredited private facility | Home | 1 | 9 | 57 | 40 |
| F | 76-85 | 2020 | Accredited private facility | Home | 1 | 7 | 0 | 15 |
| F | 76-85 | 2020 | Accredited private facility | Re-hospitalization | 1 | 7 | 50 | 38 |
| F | 76-85 | 2020 | Equivalent facility | Home | 2 | 9 | 0 | 17 |
| M | ≥ 86 | 2020 | Public facility | Home | 0 | 37 | 0 | 28 |
| F | ≥ 86 | 2020 | Accredited private facility | Home | 1 | 6 | 0 | 17 |
| F | 65-75 | 2020 | Equivalent facility | Home | 1 | 8 | 0 | 21 |
| F | 65-75 | 2020 | Public facility | Home | 1 | 12 | 0 | 23 |
| F | 76-85 | 2020 | Accredited private facility | Home | 2 | 13 | 0 | 30 |
| F | 76-85 | 2020 | Accredited private facility | Home | 1 | 5 | 0 | 4 |
| F | ≥ 86 | 2020 | Equivalent facility | Home | 2 | 10 | 0 | 41 |
| F | ≥ 86 | 2020 | Equivalent facility | Nursing home | 2 | 10 | 41 | 17 |
| M | 76-85 | 2020 | Accredited private facility | Home | 2 | 7 | 0 | 30 |
| F | ≥ 86 | 2020 | Accredited private facility | Re-hospitalization | 1 | 7 | 0 | 14 |
| F | ≥ 86 | 2020 | Accredited private facility | Home | 1 | 7 | 15 | 22 |
| F | ≥ 86 | 2020 | Accredited private facility | Home | 0 | 11 | 0 | 30 |
| F | ≥ 86 | 2020 | Accredited private facility | Death | 1 | 3 | 3 | 62 |
| M | ≥ 86 | 2020 | Equivalent facility | Home | 1 | 8 | 0 | 30 |
| F | 76-85 | 2020 | Accredited private facility | Home | 3 | 30 | 22 | 55 |
| F | 76-85 | 2020 | Equivalent facility | Home | 1 | 6 | 0 | 31 |
| M | 65-75 | 2020 | Public facility | Home | 1 | 13 | 0 | 24 |
| F | 76-85 | 2020 | Equivalent facility | Home | 7 | 14 | 0 | 52 |
| F | 76-85 | 2020 | Accredited private facility | Re-hospitalization | 1 | 5 | 0 | 1 |
| F | 76-85 | 2020 | Accredited private facility | Home | 1 | 5 | 8 | 34 |
| F | 76-85 | 2020 | Accredited private facility | Re-hospitalization | 1 | 8 | 0 | 20 |
| F | 76-85 | 2020 | Accredited private facility | Home | 1 | 8 | 212 | 47 |
| F | ≥ 86 | 2020 | Equivalent facility | Nursing home | 1 | 8 | 0 | 66 |
| M | 65-75 | 2020 | Equivalent facility | Home | 0 | 7 | 0 | 72 |
| F | 76-85 | 2020 | Public facility | Home | 3 | 12 | 0 | 14 |
| M | ≥ 86 | 2020 | Equivalent facility | Home | 2 | 7 | 0 | 30 |
| F | ≥ 86 | 2020 | Public facility | Re-hospitalization | 2 | 4 | 0 | 15 |
| F | ≥ 86 | 2020 | Accredited private facility | Home | 2 | 4 | 21 | 27 |
| F | 76-85 | 2020 | Accredited private facility | Home | 1 | 9 | 0 | 47 |
| F | ≥ 86 | 2020 | Public facility | Home | 2 | 9 | 0 | 18 |
| F | 76-85 | 2020 | Accredited private facility | Home | 1 | 10 | 70 | 29 |
| F | ≥ 86 | 2020 | Accredited private facility | Home | 5 | 34 | 44 | 28 |
| M | 76-85 | 2020 | Accredited private facility | Home | 4 | 21 | 0 | 39 |
| M | 76-85 | 2020 | Accredited private facility | Home | 4 | 21 | 39 | 16 |
| F | 76-85 | 2020 | Public facility | Home | 1 | 6 | 0 | 38 |
| F | ≥ 86 | 2020 | Equivalent facility | Home | 1 | 8 | 0 | 33 |
| F | ≥ 86 | 2020 | Equivalent facility | Re-hospitalization | 0 | 5 | 0 | 5 |
| F | ≥ 86 | 2020 | Equivalent facility | Re-hospitalization | 0 | 5 | 5 | 7 |
| F | ≥ 86 | 2020 | Equivalent facility | Re-hospitalization | 0 | 5 | 12 | 3 |
| F | ≥ 86 | 2020 | Accredited private facility | Home | 0 | 5 | 25 | 11 |
| F | 76-85 | 2020 | Equivalent facility | Home | 2 | 9 | 0 | 67 |
| F | ≥ 86 | 2020 | Equivalent facility | Re-hospitalization | 2 | 26 | 33 | 17 |
| F | ≥ 86 | 2020 | Equivalent facility | Home | 2 | 26 | 61 | 20 |
| F | ≥ 86 | 2020 | Accredited private facility | Home | 1 | 7 | 0 | 30 |
| F | 76-85 | 2020 | Accredited private facility | Home | 0 | 7 | 0 | 22 |
| F | ≥ 86 | 2020 | Accredited private facility | Re-hospitalization | 1 | 11 | 0 | 2 |
| F | ≥ 86 | 2020 | Accredited private facility | Re-hospitalization | 1 | 11 | 2 | 9 |
| F | ≥ 86 | 2020 | Accredited private facility | Home | 1 | 11 | 17 | 10 |
| F | 76-85 | 2020 | Equivalent facility | Home | 3 | 20 | 0 | 38 |
| M | ≥ 86 | 2020 | Public facility | Nursing home | 11 | 31 | 0 | 28 |
| F | 76-85 | 2020 | Public facility | Re-hospitalization | 1 | 16 | 0 | 7 |
| F | 76-85 | 2020 | Accredited private facility | Home | 6 | 14 | 0 | 41 |
| F | 76-85 | 2020 | Equivalent facility | Home | 3 | 13 | 0 | 32 |
| F | 76-85 | 2020 | Accredited private facility | Death | 1 | 16 | 0 | 20 |
| F | 65-75 | 2020 | Accredited private facility | Nursing home | 2 | 9 | 0 | 60 |
| F | ≥ 86 | 2020 | Accredited private facility | Home | 2 | 6 | 0 | 111 |
| F | ≥ 86 | 2020 | Accredited private facility | Home | 2 | 8 | 0 | 31 |
| F | ≥ 86 | 2020 | Accredited private facility | Home | 2 | 8 | 31 | 29 |
| F | ≥ 86 | 2020 | Accredited private facility | Home | 2 | 8 | 60 | 11 |
| F | ≥ 86 | 2020 | Public facility | Home | 2 | 10 | 0 | 16 |
| F | ≥ 86 | 2020 | Accredited private facility | Home | 1 | 11 | 0 | 8 |
| F | 76-85 | 2020 | Equivalent facility | Home | 3 | 7 | 0 | 25 |
| F | 76-85 | 2020 | Accredited private facility | Home | 4 | 10 | 0 | 38 |
| F | ≥ 86 | 2020 | Public facility | Nursing home | 1 | 8 | 0 | 13 |
| F | 76-85 | 2020 | Equivalent facility | Home | 2 | 13 | 0 | 16 |
| M | 76-85 | 2020 | Equivalent facility | Home | 0 | 7 | 0 | 31 |
| F | ≥ 86 | 2020 | Public facility | Home | 2 | 11 | 0 | 20 |
| F | 65-75 | 2020 | Equivalent facility | Home | 2 | 4 | 3 | 29 |
| F | ≥ 86 | 2020 | Accredited private facility | Home | 1 | 7 | 0 | 36 |
| F | 76-85 | 2020 | Equivalent facility | Home | 1 | 8 | 0 | 25 |
| F | ≥ 86 | 2020 | Accredited private facility | Home | 1 | 10 | 0 | 48 |
| F | 76-85 | 2020 | Public facility | Home | 4 | 12 | 0 | 29 |
| F | 76-85 | 2020 | Accredited private facility | Home | 0 | 9 | 0 | 29 |
| M | ≥ 86 | 2020 | Accredited private facility | Death | 1 | 14 | 0 | 8 |
| M | ≥ 86 | 2020 | Accredited private facility | Home | 2 | 16 | 0 | 29 |
| F | ≥ 86 | 2020 | Accredited private facility | Home | 4 | 6 | 21 | 60 |
| M | 76-85 | 2020 | Public facility | Home | 2 | 8 | 0 | 11 |
| M | 76-85 | 2020 | Accredited private facility | Home | 2 | 8 | 11 | 110 |
| M | 76-85 | 2020 | Accredited private facility | Death | 1 | 7 | 0 | 16 |
| F | 76-85 | 2020 | Public facility | Home | 1 | 5 | 0 | 22 |
| F | 65-75 | 2020 | Accredited private facility | Death | 3 | 10 | 0 | 14 |
| F | ≥ 86 | 2020 | Equivalent facility | Home | 1 | 8 | 0 | 48 |
| F | 76-85 | 2020 | Public facility | Home | 0 | 7 | 0 | 14 |
| F | 65-75 | 2020 | Public facility | Home | 4 | 13 | 0 | 28 |
| F | ≥ 86 | 2020 | Public facility | Re-hospitalization | 2 | 9 | 0 | 13 |
| F | ≥ 86 | 2020 | Accredited private facility | Home | 2 | 9 | 13 | 19 |
| F | 76-85 | 2020 | Public facility | Home | 2 | 12 | 0 | 27 |
| F | 76-85 | 2020 | Accredited private facility | Home | 3 | 9 | 0 | 36 |
| M | ≥ 86 | 2020 | Equivalent facility | Home | 2 | 16 | 0 | 35 |
| F | 76-85 | 2020 | Public facility | Nursing home | 3 | 10 | 0 | 37 |
| F | ≥ 86 | 2020 | Accredited private facility | Home | 0 | 6 | 0 | 40 |
| F | 76-85 | 2020 | Accredited private facility | Home | 2 | 12 | 0 | 108 |
| M | ≥ 86 | 2020 | Public facility | Nursing home | 4 | 12 | 22 | 29 |
| F | 76-85 | 2020 | Equivalent facility | Home | 2 | 9 | 0 | 40 |
| F | 76-85 | 2020 | Public facility | Home | 2 | 6 | 0 | 16 |
| F | ≥ 86 | 2020 | Accredited private facility | Death | 1 | 7 | 0 | 4 |
| M | ≥ 86 | 2020 | Accredited private facility | Home | 3 | 11 | 0 | 39 |
| M | ≥ 86 | 2020 | Accredited private facility | Home | 3 | 11 | 39 | 36 |
| F | 76-85 | 2020 | Accredited private facility | Home | 2 | 15 | 0 | 38 |
| F | 76-85 | 2020 | Accredited private facility | Home | 2 | 15 | 38 | 29 |
| F | ≥ 86 | 2020 | Accredited private facility | Home | 3 | 5 | 71 | 24 |
| F | ≥ 86 | 2020 | Equivalent facility | Home | 0 | 7 | 0 | 22 |
| F | 76-85 | 2020 | Public facility | Home | 0 | 8 | 0 | 27 |
| F | 65-75 | 2020 | Accredited private facility | Home | 3 | 14 | 0 | 25 |
| F | 65-75 | 2020 | Public facility | Home | 2 | 8 | 0 | 9 |
| F | 76-85 | 2020 | Equivalent facility | Home | 1 | 7 | 0 | 49 |
| F | ≥ 86 | 2020 | Accredited private facility | Home | 2 | 9 | 0 | 37 |
| M | 65-75 | 2020 | Equivalent facility | Home | 0 | 7 | 0 | 29 |
| F | ≥ 86 | 2020 | Accredited private facility | Home | 2 | 22 | 0 | 37 |
| F | 76-85 | 2020 | Equivalent facility | Nursing home | 1 | 8 | 0 | 35 |
| F | 76-85 | 2020 | Equivalent facility | Home | 1 | 8 | 0 | 21 |
| F | 65-75 | 2020 | Equivalent facility | Home | 2 | 8 | 0 | 41 |
| F | 76-85 | 2020 | Accredited private facility | Nursing home | 1 | 6 | 0 | 56 |
| F | 76-85 | 2020 | Accredited private facility | Re-hospitalization | 1 | 9 | 0 | 15 |
| F | ≥ 86 | 2020 | Accredited private facility | Home | 1 | 12 | 0 | 34 |
| F | 65-75 | 2020 | Accredited private facility | Home | 5 | 13 | 0 | 45 |
| F | ≥ 86 | 2020 | Public facility | Home | 1 | 25 | 137 | 28 |
| F | 76-85 | 2020 | Accredited private facility | Home | 1 | 5 | 0 | 81 |
| F | ≥ 86 | 2020 | Public facility | Home | 1 | 13 | 0 | 20 |
| F | 76-85 | 2020 | Accredited private facility | Home | 3 | 10 | 0 | 61 |
| F | 76-85 | 2020 | Accredited private facility | Nursing home | 3 | 10 | 90 | 41 |
| F | 65-75 | 2020 | Accredited private facility | Home | 1 | 6 | 0 | 30 |
| F | 76-85 | 2020 | Accredited private facility | Home | 1 | 32 | 0 | 40 |
| F | 65-75 | 2020 | Equivalent facility | Home | 2 | 8 | 0 | 38 |
| F | ≥ 86 | 2020 | Accredited private facility | Home | 3 | 11 | 0 | 30 |
| F | 76-85 | 2020 | Accredited private facility | Home | 2 | 17 | 45 | 29 |
| F | 76-85 | 2020 | Accredited private facility | Home | 0 | 11 | 0 | 45 |
| F | ≥ 86 | 2020 | Accredited private facility | Re-hospitalization | 2 | 8 | 0 | 18 |
| F | ≥ 86 | 2020 | Equivalent facility | Nursing home | 2 | 8 | 60 | 4 |
| F | ≥ 86 | 2020 | Accredited private facility | Home | 47 | 54 | 0 | 55 |
| F | 76-85 | 2020 | Public facility | Re-hospitalization | 4 | 33 | 0 | 7 |
| F | 76-85 | 2020 | Public facility | Home | 4 | 33 | 13 | 7 |
| F | 65-75 | 2020 | Public facility | Re-hospitalization | 8 | 17 | 0 | 53 |
| F | 65-75 | 2020 | Accredited private facility | Home | 8 | 17 | 53 | 31 |
| F | ≥ 86 | 2020 | Accredited private facility | Nursing home | 1 | 14 | 0 | 27 |
| F | ≥ 86 | 2020 | Accredited private facility | Nursing home | 1 | 19 | 0 | 40 |
| M | ≥ 86 | 2020 | Accredited private facility | Home | 0 | 4 | 0 | 28 |
| F | 76-85 | 2020 | Public facility | Home | 1 | 8 | 0 | 28 |
| F | ≥ 86 | 2020 | Public facility | Home | 4 | 11 | 0 | 24 |
| F | 76-85 | 2020 | Equivalent facility | Home | 2 | 9 | 0 | 30 |
| F | 65-75 | 2020 | Accredited private facility | Home | 1 | 8 | 0 | 33 |
| M | ≥ 86 | 2020 | Equivalent facility | Re-hospitalization | 2 | 9 | 47 | 28 |
| F | ≥ 86 | 2020 | Public facility | Home | 2 | 6 | 0 | 16 |
| M | 76-85 | 2020 | Public facility | Home | 4 | 12 | 0 | 28 |
| F | 76-85 | 2020 | Equivalent facility | Home | 3 | 18 | 0 | 41 |
| F | ≥ 86 | 2020 | Accredited private facility | Home | 4 | 10 | 0 | 45 |
| M | 65-75 | 2020 | Accredited private facility | Home | 0 | 7 | 0 | 15 |
| F | 65-75 | 2020 | Public facility | Home | 1 | 5 | 0 | 26 |
| F | 76-85 | 2020 | Public facility | Home | 2 | 9 | 0 | 14 |
| F | ≥ 86 | 2020 | Public facility | Home | 2 | 6 | 0 | 21 |
| M | 65-75 | 2020 | Accredited private facility | Home | 1 | 5 | 0 | 12 |
| F | 76-85 | 2020 | Public facility | Home | 2 | 4 | 0 | 32 |
| F | 65-75 | 2020 | Accredited private facility | Home | 0 | 17 | 0 | 102 |
| F | ≥ 86 | 2020 | Equivalent facility | Home | 1 | 8 | 0 | 24 |
| F | 65-75 | 2020 | Accredited private facility | Home | 13 | 21 | 0 | 37 |
| F | 65-75 | 2020 | Accredited private facility | Home | 2 | 8 | 0 | 38 |
| F | 76-85 | 2020 | Accredited private facility | Home | 2 | 7 | 0 | 39 |
| F | 76-85 | 2020 | Equivalent facility | Home | 1 | 7 | 0 | 53 |
| F | ≥ 86 | 2020 | Accredited private facility | Home | 1 | 18 | 0 | 84 |
| F | 76-85 | 2020 | Accredited private facility | Home | 2 | 15 | 0 | 41 |
| F | ≥ 86 | 2020 | Public facility | Home | 11 | 17 | 0 | 30 |
| F | 65-75 | 2020 | Public facility | Home | 1 | 8 | 0 | 3 |
| F | 65-75 | 2020 | Accredited private facility | Home | 1 | 15 | 0 | 36 |
| F | ≥ 86 | 2020 | Public facility | Home | 1 | 7 | 0 | 23 |
| F | 76-85 | 2020 | Equivalent facility | Re-hospitalization | 3 | 11 | 0 | 49 |
| F | ≥ 86 | 2020 | Accredited private facility | Home | 2 | 12 | 0 | 46 |
| F | 76-85 | 2020 | Accredited private facility | Re-hospitalization | 0 | 8 | 0 | 7 |
| F | 76-85 | 2020 | Accredited private facility | Home | 0 | 8 | 7 | 30 |
| F | 76-85 | 2020 | Accredited private facility | Re-hospitalization | 2 | 10 | 0 | 11 |
| F | 76-85 | 2020 | Accredited private facility | Re-hospitalization | 2 | 10 | 13 | 13 |
| F | 76-85 | 2020 | Accredited private facility | Home | 2 | 10 | 26 | 19 |
| M | 65-75 | 2020 | Accredited private facility | Home | 1 | 8 | 0 | 32 |
| M | 65-75 | 2020 | Public facility | Home | 2 | 5 | 0 | 24 |
| F | ≥ 86 | 2020 | Public facility | Home | 2 | 8 | 0 | 35 |
| F | 76-85 | 2020 | Accredited private facility | Home | 1 | 7 | 0 | 30 |
| F | ≥ 86 | 2020 | Accredited private facility | Nursing home | 1 | 19 | 0 | 43 |
| M | ≥ 86 | 2020 | Public facility | Re-hospitalization | 4 | 25 | 0 | 17 |
| M | ≥ 86 | 2020 | Accredited private facility | Home | 4 | 25 | 18 | 28 |
| F | ≥ 86 | 2020 | Accredited private facility | Home | 1 | 6 | 0 | 40 |
| M | 65-75 | 2020 | Equivalent facility | Home | 2 | 15 | 0 | 21 |
| F | 65-75 | 2020 | Equivalent facility | Home | 1 | 6 | 0 | 30 |
| M | 65-75 | 2020 | Accredited private facility | Home | 1 | 7 | 0 | 27 |
| F | 76-85 | 2020 | Accredited private facility | Home | 3 | 10 | 0 | 30 |
| F | ≥ 86 | 2020 | Accredited private facility | Home | 1 | 13 | 0 | 30 |
| F | ≥ 86 | 2020 | Accredited private facility | Home | 1 | 13 | 203 | 47 |
| F | ≥ 86 | 2020 | Accredited private facility | Re-hospitalization | 1 | 22 | 0 | 40 |
| F | ≥ 86 | 2020 | Accredited private facility | Home | 1 | 22 | 40 | 11 |
| F | ≥ 86 | 2020 | Equivalent facility | Home | 0 | 9 | 0 | 27 |
| F | 76-85 | 2020 | Accredited private facility | Home | 1 | 11 | 0 | 17 |
| F | 76-85 | 2020 | Accredited private facility | Home | 1 | 13 | 0 | 28 |
| M | 76-85 | 2020 | Accredited private facility | Home | 0 | 20 | 0 | 36 |
| F | 76-85 | 2020 | Public facility | Re-hospitalization | 1 | 5 | 99 | 13 |
| F | 76-85 | 2020 | Accredited private facility | Home | 1 | 5 | 112 | 58 |
| F | 76-85 | 2020 | Equivalent facility | Home | 0 | 10 | 0 | 37 |
| F | 76-85 | 2020 | Equivalent facility | Home | 3 | 11 | 0 | 42 |
| F | 76-85 | 2020 | Accredited private facility | Home | 0 | 5 | 0 | 10 |
| F | 76-85 | 2020 | Public facility | Re-hospitalization | 1 | 7 | 0 | 28 |
| F | 76-85 | 2020 | Accredited private facility | Home | 1 | 7 | 28 | 21 |
| M | ≥ 86 | 2020 | Public facility | Re-hospitalization | 0 | 7 | 0 | 15 |
| M | ≥ 86 | 2020 | Accredited private facility | Home | 0 | 7 | 15 | 19 |
| F | ≥ 86 | 2020 | Equivalent facility | Home | 3 | 9 | 0 | 30 |
| M | ≥ 86 | 2020 | Equivalent facility | Home | 2 | 7 | 0 | 145 |
| M | 76-85 | 2020 | Equivalent facility | Home | 2 | 7 | 0 | 30 |
| F | 76-85 | 2020 | Public facility | Home | 1 | 15 | 0 | 16 |
| F | ≥ 86 | 2020 | Accredited private facility | Home | 4 | 12 | 0 | 30 |
| F | 65-75 | 2020 | Accredited private facility | Home | 2 | 7 | 110 | 13 |
| F | 65-75 | 2020 | Public facility | Home | 2 | 7 | 132 | 16 |
| F | 76-85 | 2020 | Equivalent facility | Home | 4 | 8 | 0 | 45 |
| F | 65-75 | 2020 | Public facility | Home | 1 | 10 | 0 | 28 |
| M | 65-75 | 2020 | Equivalent facility | Re-hospitalization | 3 | 7 | 0 | 12 |
| M | 65-75 | 2020 | Equivalent facility | Home | 3 | 7 | 37 | 25 |
| M | ≥ 86 | 2020 | Accredited private facility | Home | 1 | 8 | 0 | 38 |
| F | ≥ 86 | 2020 | Public facility | Nursing home | 0 | 10 | 0 | 16 |
| M | 65-75 | 2020 | Public facility | Home | 3 | 17 | 0 | 14 |
| F | ≥ 86 | 2020 | Accredited private facility | Home | 4 | 11 | 0 | 93 |
| M | 65-75 | 2020 | Public facility | Re-hospitalization | 1 | 10 | 0 | 8 |
| F | 76-85 | 2020 | Public facility | Home | 1 | 8 | 0 | 29 |
| F | 65-75 | 2020 | Accredited private facility | Re-hospitalization | 2 | 10 | 0 | 37 |
| F | 65-75 | 2020 | Accredited private facility | Home | 2 | 10 | 45 | 46 |
| F | 65-75 | 2020 | Public facility | Home | 2 | 4 | 0 | 14 |
| M | 65-75 | 2020 | Public facility | Home | 1 | 12 | 0 | 17 |
| M | 76-85 | 2020 | Accredited private facility | Re-hospitalization | 1 | 4 | 41 | 41 |
| M | 76-85 | 2020 | Accredited private facility | Nursing home | 1 | 4 | 82 | 16 |
| M | ≥ 86 | 2020 | Accredited private facility | Home | 1 | 8 | 0 | 4 |
| F | 76-85 | 2020 | Equivalent facility | Home | 2 | 9 | 0 | 19 |
| M | 76-85 | 2020 | Public facility | Re-hospitalization | 0 | 11 | 0 | 6 |
| M | 76-85 | 2020 | Accredited private facility | Re-hospitalization | 0 | 11 | 62 | 17 |
| M | 76-85 | 2020 | Accredited private facility | Nursing home | 0 | 11 | 79 | 56 |
| F | 65-75 | 2020 | Accredited private facility | Re-hospitalization | 3 | 19 | 0 | 23 |
| F | 65-75 | 2020 | Accredited private facility | Nursing home | 3 | 19 | 27 | 79 |
| F | 76-85 | 2020 | Accredited private facility | Home | 1 | 11 | 0 | 57 |
| F | 76-85 | 2020 | Accredited private facility | Death | 1 | 7 | 0 | 18 |
| F | 65-75 | 2020 | Public facility | Home | 3 | 8 | 0 | 27 |
| F | 76-85 | 2020 | Accredited private facility | Re-hospitalization | 0 | 14 | 0 | 19 |
| M | 65-75 | 2020 | Accredited private facility | Re-hospitalization | 1 | 8 | 0 | 35 |
| M | 65-75 | 2020 | Accredited private facility | Re-hospitalization | 1 | 8 | 35 | 39 |
| F | ≥ 86 | 2020 | Public facility | Home | 0 | 8 | 0 | 22 |
| F | 65-75 | 2020 | Accredited private facility | Home | 3 | 14 | 0 | 37 |
| F | 76-85 | 2020 | Public facility | Re-hospitalization | 1 | 53 | 0 | 13 |
| F | 76-85 | 2020 | Accredited private facility | Home | 1 | 53 | 21 | 45 |
| F | 76-85 | 2020 | Accredited private facility | Home | 1 | 53 | 66 | 12 |
| M | 65-75 | 2020 | Public facility | Home | 0 | 3 | 0 | 13 |
| F | 76-85 | 2020 | Equivalent facility | Home | 2 | 15 | 0 | 29 |
| F | ≥ 86 | 2020 | Accredited private facility | Home | 1 | 8 | 0 | 40 |
| F | ≥ 86 | 2020 | Accredited private facility | Home | 1 | 8 | 40 | 25 |
| F | 76-85 | 2020 | Public facility | Re-hospitalization | 2 | 8 | 0 | 15 |
| M | 65-75 | 2020 | Accredited private facility | Home | 1 | 7 | 0 | 25 |
| F | 65-75 | 2020 | Accredited private facility | Home | 0 | 11 | 0 | 27 |
| F | 65-75 | 2020 | Equivalent facility | Home | 0 | 11 | 141 | 28 |
| F | 65-75 | 2020 | Equivalent facility | Home | 3 | 18 | 0 | 25 |
| F | 65-75 | 2020 | Equivalent facility | Home | 4 | 8 | 0 | 28 |
| F | ≥ 86 | 2020 | Public facility | Nursing home | 2 | 6 | 0 | 27 |
| F | 65-75 | 2020 | Accredited private facility | Home | 2 | 14 | 0 | 30 |
| M | 76-85 | 2020 | Equivalent facility | Home | 4 | 13 | 0 | 6 |
| M | 65-75 | 2020 | Accredited private facility | Home | 1 | 7 | 0 | 16 |
| F | 65-75 | 2020 | Accredited private facility | Home | 4 | 12 | 0 | 30 |
| F | 76-85 | 2020 | Accredited private facility | Home | 1 | 4 | 8 | 40 |
| M | 65-75 | 2020 | Accredited private facility | Home | 3 | 42 | 0 | 96 |
| F | 65-75 | 2020 | Accredited private facility | Home | 1 | 6 | 0 | 31 |
| M | ≥ 86 | 2020 | Accredited private facility | Nursing home | 0 | 9 | 0 | 50 |
| F | ≥ 86 | 2020 | Equivalent facility | Home | 2 | 10 | 0 | 30 |
| F | 65-75 | 2020 | Equivalent facility | Home | 0 | 8 | 0 | 23 |
| F | ≥ 86 | 2020 | Accredited private facility | Re-hospitalization | 0 | 9 | 9 | 18 |
| F | ≥ 86 | 2020 | Accredited private facility | Nursing home | 0 | 9 | 27 | 33 |

*Step 1: Average time in days between hospital admission and surgery. Step 2: Average time in days for the hospital stay. Step 3: Average time in days between hospital discharge and rehabilitation facility admission. Step 4: Average time in days for the rehabilitation. Abbreviations: F, Female; M, Male.*
